# Environmental Association Analyses Identify Candidates for Abiotic Stress Tolerance in *Glycine soja*, the Wild Progenitor of Cultivated Soybeans

**DOI:** 10.1534/g3.116.026914

**Published:** 2016-01-27

**Authors:** Justin E. Anderson, Thomas J. Y. Kono, Robert M. Stupar, Michael B. Kantar, Peter L. Morrell

**Affiliations:** * Department of Agronomy and Plant Genetics, University of Minnesota, St. Paul, Minnesota 55108; † Biodiversity Research Centre and Department of Botany, University of British Columbia, Vancouver, British Columbia V6T 1Z4, Canada

**Keywords:** crop wild relative, landscape genomics, germplasm collections, population structure, soybean, *Glycine soja*

## Abstract

Natural populations across a species range demonstrate population structure owing to neutral processes such as localized origins of mutations and migration limitations. Selection also acts on a subset of loci, contributing to local adaptation. An understanding of the genetic basis of adaptation to local environmental conditions is a fundamental goal in basic biological research. When applied to crop wild relatives, this same research provides the opportunity to identify adaptive genetic variation that may be used to breed for crops better adapted to novel or changing environments. The present study explores an *ex situ* conservation collection, the USDA germplasm collection, genotyped at 32,416 SNPs to identify population structure and test for associations with bioclimatic and biophysical variables in *Glycine soja*, the wild progenitor of *Glycine max* (soybean). Candidate loci were detected that putatively contribute to adaptation to abiotic stresses. The identification of potentially adaptive variants in this *ex situ* collection may permit a more targeted use of germplasm collections.

Local adaptation has been a topic of research for more than a century and a half. Wallace (1858) observed that organisms in different environments are phenotypically distinct. Despite the importance of local environments, historically, it has been difficult to assess response to environmental conditions across entire species ranges, and to disentangle the effects of selection from demography (Sexton *et al.* 2009). This, in turn, has made it problematic to use species range data in concert with crop germplasm collections and breeding programs. Global biophysical and bioclimatic data along with genome-wide marker genotypes creates the opportunity to use a ‘landscape genomics’ framework to potentially uncover the genetic basis for adaptive differences among populations (reviewed in Bragg *et al.* 2015). The application of ‘landscape genomics’ to crop wild relatives is particularly compelling, as the variation identified may then be tested for targeted crop improvement. As cultivars are typically derived from a subset of wild progenitors (Harlan *et al.* 1973), studies of crop wild relatives have the potential to uncover adaptive variants that do not occur in current cultivars.

Several approaches have been developed to identify genetic variants that may contribute to local adaptation. Lewontin and Krakauer (1973) proposed comparisons of subpopulations to identify loci with large allele frequency differences as measured by *F*_ST_ (Wright 1949). Simulation studies suggest the Lewontin and Krakauer framework provides a useful means of identifying potentially adaptive variants (Beaumont and Balding 2004; Beaumont 2005). Newer approaches identify allele frequency gradients or use mixed model approaches to identify an association between genetic variants and environmental variables. For example, Spatial Ancestry Analysis (SPA) (Yang *et al.* 2012) is appropriate for sampling of individuals across continuous geographic space and environmental gradients. SPA estimates a continuous function of allele frequency that can be projected onto geographic space. Loci showing steep gradients in allele frequency are inferred to have been subject to selection (Yang *et al.* 2012). Mixed model association mapping, as used in studies of phenotypic variation (Lipka *et al.* 2015), can also be used to associate genetic variants with environmental variables (Eckert *et al.* 2010; Pyhäjärvi *et al.* 2013; Yoder *et al.* 2014).

The potential to identify genetic variants that contribute to local adaptation in crop wild relatives has important agricultural implications. Crop wild relatives are often a source of novel variation for plant breeding (Khoury *et al.* 2010; McCouch *et al.* 2013). To date, introgression of adaptive variation from wild relatives has typically involved crosses with single accessions containing favorable characteristics (*e.g.*, resistance to a particular pathogen). Screening an entire germplasm collection for agronomically important traits is impractical for individual breeding programs. Environmental association within *ex situ* germplasm collections provides a path to targeted use of crop wild relatives.

*Glycine soja*, the wild progenitor of cultivated soybean, is native to East Asia, with a broad ecological distribution in China, Japan, Korea, and Russia (Li *et al.* 2010). Environmental variation is extensive across its range, with altitude ranging from sea level to ∼1400 m, yearly precipitation ranging from 300 mm to 3400 mm, and mean annual temperature ranging from –3.1° to 18.2°. The ecological amplitude of many crop wild relatives is very different from that observed for the crop in cultivation (Khoury *et al.* 2015). For soybean, a large portion of production (including a major growing region in the United States), occurs in an environmentally and climatically similar region to the natural range of the wild progenitor. In the United States, soybean is cultivated in areas with altitude ranging from sea level to ∼900 m, yearly precipitation ranging from 400 mm to 1800 mm, and mean annual temperature ranging from 1.3° to 20.5°. Given this environmental similarity, and the ease of crossing of *Glycine soja* with cultivated soybean, variants identified can be readily tested in soybean breeding programs.

In this study, we examined population structure, environmental associations, and allele frequency gradients in 533 accessions of *Glycine soja*. The sampled accessions are derived from the USDA GRIN soybean germplasm collection, and were genotyped with the SoySNP50K genotyping platform (Song *et al.* 2015; GRIN 2015). Environmental association was performed using data from public repositories of global bioclimatic (WorldClim) (Hijmans *et al.* 2005), and biophysical (soils) variables (ISRIC) (Hengl *et al.* 2014) with 1 km resolution. Bioclimatic data include a summary of precipitation and temperature variables, which constitute averages over a period of 50 yr. Biophysical data include soil texture and chemistry variables. Environmental association, SPA, and *F*_ST_ outliers were explored, identifying variants that may be useful in targeted improvement of abiotic stress tolerance in soybean.

## Materials and Methods

### Genetic data acquisition

**Figure 1:**
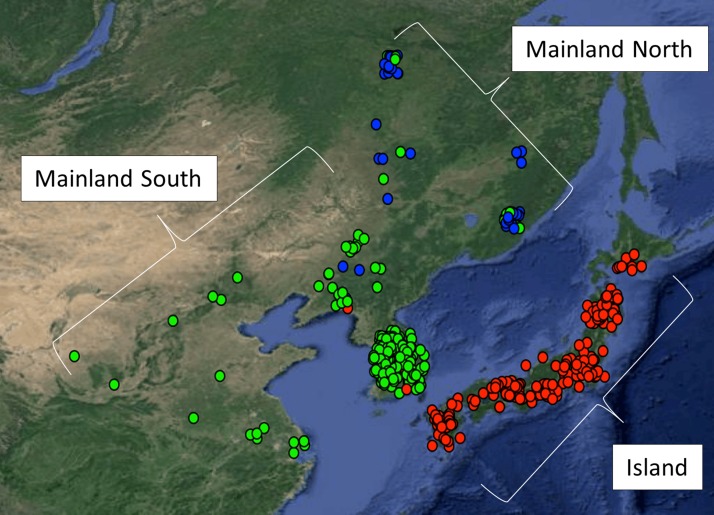
Results of STRUCTURE analysis in *G. soja* accessions, and the geographical location in which each were collected. The spot colors correspond to the STRUCTURE assignment of each accession, Green: Mainland South; Blue: Mainland North; Red: Island. The assignment of samples into three genetic clusters generally accords with geography. The spots have been jittered to show overlapping samples.

Genotype data from the SoySNP50K platform (Song *et al.* 2015) were downloaded from SoyBase (Grant *et al.* 2010) for all available *G. soja* accessions. We excluded accessions without latitude and longitude coordinates, those with > 10% missing data, duplicate accessions (based on genetic similarity of ≥99.9%). We also excluded eight accessions from Taiwan and Northern Russia that were geographic outliers. Ambiguous and heterozygous SNP calls were treated as missing data due to the low outcrossing rate (∼3%) in *G. soja* (Kuroda *et al.* 2006; Guo *et al.* 2012). A list of the accession (Plant Introduction or PI) numbers, and geographic origins of the *G. soja* accessions used in this study is available in Supporting Information, Table S1, and a map of our sampled accessions is shown in Figure 1. The physical map positions of the SoySNP50K SNPs (Song *et al.* 2013) were mapped into the second genome assembly ‘Glyma.Wm82.a2.v1’ (http://www.soybase.org/), where the SNP query sequences were aligned with Bowtie 2 (Langmead and Salzberg 2012). The resulting SAM (Li *et al.* 2009) file was parsed with a custom Python script to extract the SNP position on the version 2 assembly. A translation table of the SoySNP50K SNPs, from version 1 assembly positions ‘Glyma.Wm82.a1’ (Schmutz *et al.* 2010) to version 2 assembly positions ‘Glyma.Wm82a2’ (Song *et al.* 2016), is available in Table S2.

### Bioclimatic and biophysical variables

The latitude and longitude coordinates of *G. soja* sampling locations were used to query the WorldClim database for 68 variables, including bioclimatic variables based on yearly, quarterly, and monthly temperature and precipitation data, as well as altitude data at a resolution of 30 arcseconds (approximately 1 km grids) (Table S3; Hijmans *et al.* 2005). The sampling locations (longitude and latitude) were also used to query the ISRIC database (World Soil Information database; Hengl *et al.* 2014) for seven biophysical variables [pH × 10 in H_2_O, percentage sand, percentage silt, percentage clay, bulk density in kg/m^3^, cation exchange capacity in cmolc/kg, and organic carbon content (fine earth fraction) in permilles] at a resolution of 30 arcseconds (Table S3). We grouped soil data into two classes: topsoil (from 0 cm to 30 cm), and subsoil (from 30 cm to 200 cm), resulting in 14 soil variables. Classes were created by averaging the appropriate depths from the six depths available in the ISRIC database: 2.5 cm, 10 cm, 22.5 cm, 45 cm, 80 cm, and 150 cm (Hengl *et al.* 2014). Principal component analysis (PCA) on the bioclimatic and biophysical variables (first scaled to a mean of 0 and standard deviation of 1) was conducted using the prcomp function in the R statistical language and programming environment (R Development Core Team 2015). Pearson correlation coefficients between bioclimatic and biophysical variables were calculated in R (Table S4). Boxplots for each scaled bioclimatic and biophysical variable were created based on *G. soja* localities to examine the distribution for each variable (Figure S1).

### Population structure, allelic composition, and linkage disequilibrium measures

Genetic assignment analysis was used to identify population structure in the sample using a Bayesian Monte Carlo Markov Chain (MCMC) algorithm implemented in STRUCTURE (Pritchard *et al.* 2000). The number of clusters (*K*), ranging from 2 to 5, was explored using a model with uncorrelated allele frequencies and no admixture between clusters, parameters that reflect a high degree of observed allele frequency differentiation among populations, and no prior evidence of admixture in *G. soja*. Runs for each *K* value were replicated 10 times, with 10,000 burn-in steps, and recorded for 10,000 subsequent steps. STRUCTURE assignments were visualized with the CLUMPPAK server (Kopelman *et al.* 2015). PCA was also used to explore population structure using the SNPRelate package (Figure S2) (Zheng *et al.* 2012).

*F*_ST_ was estimated among populations identified through genetic assignment (STRUCTURE). Theta (Ɵ), the variance-based *F*_ST_ estimate of Weir and Cockerham (1984), was estimated using the R ’hierfstat’ package (Goudet 2005). The private allele richness of populations was calculated with the rarefaction approach implemented in ADZE to account for differences in sample size (Szpiech *et al.* 2008). For visualization, *F*_ST_ was averaged in sliding windows, with a window size of 5, and a step of 3 SNPs. A Mantel test was conducted to explore isolation by distance, utilizing great circle distance between geographic locations, and pairwise genetic distance using the ‘vegan’ package in R (Oksanen *et al.* 2007).

SPA was used to detect loci showing steep gradients in allele frequency (Yang *et al.* 2012). SNPs with SPA scores above the 99.9th percentile were identified as outliers. SPA comparisons are particularly compelling for species with a continuous distribution and relationship among individuals driven by isolation by distance, as it incorporates geographic and genetic gradients in identifying local clines (Yang *et al.* 2012).

Linkage disequilibrium (LD) in the sample was calculated with the ‘LDheatmap’ package in R (Shin *et al.* 2006). LD as D′ (Lewontin 1964) was calculated between all pairwise combinations of markers on each chromosome. LD decay over physical distance was estimated using the exponential regression method of Abecasis *et al.* (2001). Because recombination rate differed strongly between pericentromeric regions and euchromatic regions (Lee *et al.* 2015), we treated these regions separately. For calculating the decay curves, we used 2.39 cM/Mb and 3.59 cM/Mb for pericentromeric and euchromatic regions, respectively, based on median adjacent-SNP recombination rate from the genetic map of Lee *et al.* (2015).

### Environmental association mapping

Mixed-model association as implemented in Tassel (5.0v) (Bradbury *et al.* 2007; Zhang *et al.* 2010) was used to test for associations between individual SNPs and bioclimatic and biophysical variables. The following models were explored: a naïve model with no control for population structure, a model using the *Q*-matrix from STRUCTURE to control for population structure, a model using a kinship matrix (*K*-matrix) based on pairwise Manhattan distance among individuals at all SNPs, a model using both a *K*-matrix and a *Q*-matrix, and models that integrate latitude or latitude and longitude as covariates. Quantile-Quantile (qq) plots were examined for each model, and the genomic inflation parameter lambda (Λ) (Devlin and Roeder 1999), a metric of *p*-value inflation, was calculated (Figure S3).

A threshold set at the 0.01% most extreme *p*-values was used to identify candidates for each environmental association, resulting in three significant marker associations per environmental variable. This threshold was chosen to focus the analysis on a minimum number of large effect variants, and to limit false positives. The qqman R package (Turner 2014) was used to plot the association results.

### Candidate characterization

SNPs identified as outliers through the environmental association mapping, SPA, or *F*_ST_ approaches were examined for functional annotation using SoyBase (www.soybase.org) (Grant *et al.* 2010). This database provided access to minor allele frequency (MAF) for panels of landraces, elite lines, and *G. soja* accessions, based on data from the SoySNP50K development study (Song *et al.* 2013). For each SNP outlier, we collected additional information on genic context, nearby annotated genes, and the inferred Arabidopsis ortholog (TAIR10 best hit provided by Soybase). We performed enrichment analysis to determine if euchromatin, 3′ UTR, 5′ UTR, coding sequence (CDS), and intronic regions we over or underrepresented among outliers. Significance of enrichment was assessed by creating a 99% confidence interval around the proportion of SNPs that was found in each category as calculated by bootstrap sampling the number of SNPs in each category 1000 times.

### Data availability

The scripts and most of the input files used to filter SNPs, run STRUCTURE, calculate *F*_ST_, calculate LD, and generate figures are publicly available in the GitHub repository located at https://github.com/MorrellLAB/Soja_Env_Association. A publicly available version of the genotyping matrix is available at (http://soybase.org/snps/index.php).

## Results

### Population structure

The filtering criteria resulted in 533 *G. soja* accessions for analysis from the GRIN collection. These accessions came from 273 unique sampling locations (Figure 1). Each accession was genotyped at 32,416 polymorphic SNPs that remained from SoySNP50K (Song *et al.* 2015) after filtering. These SNPs were distributed throughout the euchromatic and pericentromeric regions, and spaced at an average of ∼8.6 kb and ∼45 kb, respectively. Genetic assignment was assessed at *K* = 2 to *K* = 5. At *K* = 2 accessions were divided primarily east and west of the Sea of Japan. At *K* = 3, the Japanese archipelago samples form a distinct cluster, and the mainland samples were split into a northern and southern cluster. With *K* = 4, the samples located on the Korean Peninsula began to separate from mainland Asia, forming a unique cluster, or, more infrequently, the Japanese samples subdivided into two clusters. For *K* = 5, the Japan cluster separated into two distinct northern and southern subpopulations. Most analyses reported here are based on *K* = 3, which was identified as the optimum number of clusters using the approach of Evanno *et al.* (2005). The three clusters are identified here as Island (Japan), Mainland North (Northeast China and Eastern Russia), and Mainland South (Eastern China and South Korea) (Figure 1). The clustering corresponds primarily to physical barriers to migration, and accords well with previously published studies of population structure in *G. soja* (Kuroda *et al.* 2006; Kaga *et al.* 2012; Guo *et al.* 2012). PCA of the genetic data identified a similar pattern of genetic clustering (Figure S2). The first principal component (PC) explained 5.1% of the variation, and primarily separated samples on an east–west gradient. The second PC explained 2.7% of the variation, and a largely north–south gradient. A Mantel test identified isolation by distance in our sample (*r* = 0.58, *p* < 0.001).

### Allele frequency differentiation, pairwise diversity, and linkage disequilibrium

The Island and Mainland South clusters include the most accessions, at 216 and 275, respectively. The Mainland North cluster was smaller, with 42 individuals. *Glycine soja* had a mean pairwise similarity of ∼70% across all samples (Figure S4A). The Mainland North population was an outlier in terms of mean pairwise similarity within the three clusters, and contained a smaller number of segregating sites (Table 1 and Figure S4B). The Island cluster had the highest private allele richness (corrected for sample size), followed by the Mainland South, then Mainland North (Table 1). There were no fixed differences between populations. Among these three groups, the genome-wide average per SNP *F*_ST_ = 0.1. The genome-wide average Mainland South by Mainland North *F*_ST_ = 0.11, Mainland South by Island *F*_ST_ = 0.07, and Mainland North by Island *F*_ST_ = 0.18. The average half-life of LD in the euchromatic regions was D′ = 34 kb, substantially lower than the pericentromeric regions, with an average half-life of D′ = 500 kb. This was similar to previously reported values for *G. soja* (Zhou *et al.* 2015). Heatmaps of LD and curves of LD decay over distance are shown in Figure S5.

**Table 1:** Diversity summary statistics within assigned clusters of *Glycine soja* sampled

Population	Sample Size	Segregating Sites	Private Allele Richness	Mean Pairwise Difference
Island	216	31,698	0.025 (0.011)	0.340
Mainland South	275	32,360	0.009 (0.005)	0.337
Mainland North	42	23,797	0.001 (0.0001)	0.306
Mainland South + Island	492	32,416	0.250 (0.160)	0.349
Mainland North + Island	258	32,350	0.006 (0.002)	0.345
Mainland South + Mainland North	317	32,360	0.045 (0.029)	0.338

Data include sample size, number of segregating sites, private allele richness (expected number of private alleles adjusted for sample size) with standard deviation, Mean Pairwise Difference (average difference between two accessions in each cluster).

### Environmental variability and interdependence

Most of the 82 environmental variables showed a high degree of variation across the 273 sampled locations (Figure S1). Across the range of *G. soja*, the northwestern region is colder and drier (Figure S6A and Figure S6B). The soils data indicate that the portion of the range in Japan has lower organic matter, higher sand content, and more variable pH than the mainland (Figure S6C and Table S3). A PCA of the bioclimatic and biophysical variables recapitulates the geography (Figure S6D). The first four PCs explained 86.3% of the variation. Specifically, the first PC was associated with temperature, the second PC with precipitation seasonality (coefficient of variation in yearly precipitation), the third PC precipitation/soil, and the fourth PC with soil. Pearson correlations between all bioclimatic and biophysical variables (Table S4) showed high correlation between topsoil and subsoil (> 0.99); temperature of adjacent months (> 0.91); and spring, summer, and fall precipitation. While precipitation in July and August was highly correlated (0.86), they had low correlation with adjacent months (June–July precipitation = 0.36; August–September precipitation = 0.18).

### Environmental association mapping

Environmental association mapping using mixed models that incorporated the *K*-matrix and *Q* + *K* matrix outperformed a naïve model or Q-matrix only model (Figure S3). The final model used the bioclimatic/biophysical variable as the response, and genotype as a fixed effect, the *K*-matrix as a random effect, and latitude as a covariate (Figure S3). This was the simplest model (fewest covariates), with a Λ near 1, indicating minimal inflation of *p*-values (Figure S3). The use of latitude, or latitude and longitude, as covariates resulted in nearly the same Λ.

This model was applied across all 82 environmental variables. All *p*-values from mixed model analyses are reported in Table S5 (Benjamini-Hochberg FDR-values in Table S6). As expected with a marker set this size, typical thresholds of 0.01 Benjamini-Hochberg FDR-value, or 0.001 *p*-value, resulted in thousands of markers below the significance threshold (on average 1159 or 77 markers per environmental variable association). These thresholds were therefore deemed insufficient for extracting only major loci. Instead, the threshold of 0.01% for each association was investigated, corresponding to the three strongest marker associations for each bioclimatic and biophysical variable. At this significance level, 110 unique SNPs were associated with at least one bioclimatic or biophysical variable (Table S7). The *p*-value for these significant SNPs ranged from 4.05E–4 to 5.69E–9, with an average of 2.98E–5. We examined GO terms and the putative function of *Arabidopsis thaliana* orthologs for genes within 34 kb (average euchromatic half-life of LD in our sample) of these significant markers. As expected, several patterns arose corresponding to correlated environmental variables (Table S4), and major contributors to the environmental PCA (Figure S6D).

**Figure 2:**
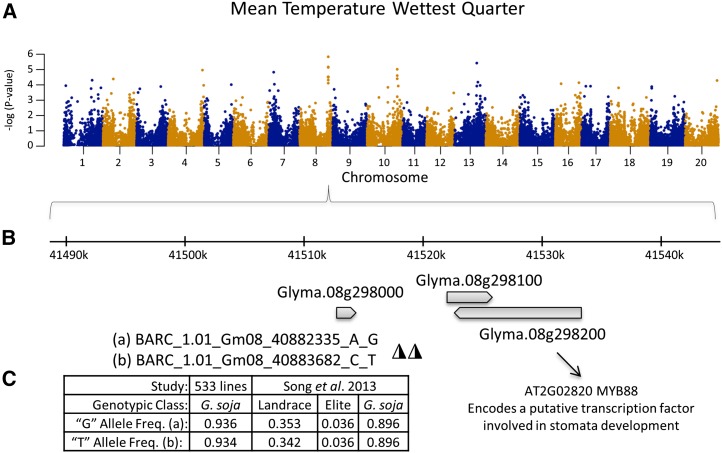
Genome-wide associations with Mean Temperature Wettest Quarter. (A) Manhattan plot of negative log *p*-values. (B) Zoom in on 60 kb region around the significant markers BARC_1.01_Gm08_40882335_A_G and BARC_1.01_Gm08_40883682_C_T. The Arabidopsis ortholog for a nearby gene, Glyma.08g298200, is MYB88, a gene-associated stomata development. (C) The frequency of nonreference “G” and “T” alleles is high in *G. soja*, and rare in a previous study of landrace and elite lines. (Note: The marker name is based on the genome assembly version 1 position (e.g., 40882335) while the position shown in (B) is based on the assembly version 2 position (e.g., approximately 41520000). This explanation also applies to the discrepancy between marker name and genome position in Figures 3 and 4.)

Mean Temperature Wettest Quarter was a major contributor to PC1 of the environmental PCA. Mixed model association of this variable identified an association on chromosome 8 with two SNPs (*p* = 1.47E–6 and 6.78E–6) occurring less than 5 kb downstream of Glyma.08g298200 (Figure 2, A and B). The Arabidopsis ortholog (as inferred from Soybase) is MYB88, functionally annotated as “Encodes a putative transcription factor involved in stomata development.” The nonreference alleles for the two significant markers at this locus are more common in *G. soja* than in landrace and elite soybean lines (Figure 2C). The environmental trait distribution of Mean Temperature Wettest Quarter reveals that, while both the reference and nonreference alleles are found in all three population clusters (Figure S7A), individuals with the nonreference allele occur in environments that are on average ∼2° warmer in the wettest quarter than those with the reference variant (Figure S7B).

Often, the same genetic variants were found associated with more temperature variables from adjacent months. An example is SNP ‘BARC_1.01_Gm16_1552499_A_G,’ significant in 11 associations with summer temperature variables (Figure S8). Many of these bioclimatic variables were major contributors to PC1 (temperature) in the environmental PCA. The Arabidopsis ortholog for the nearest gene, Glyma.16g017600, was TMP14, which encodes the P subunit of Photosystem I (Khrouchtchova *et al.* 2005). Individuals with the reference variant tended to occur at sites with cooler summer temperatures (Figure S9).

One of the strongest associations with monthly precipitation was between SNP ‘BARC_1.01_Gm_08_2254106_G_A,’ on chromosome 8, and July Precipitation (Figure S10). This SNP was significant in both July Precipitation, and Precipitation Wettest Quarter. This SNP occurs within Glyma.08g028200 (Figure S10). The Arabidopsis ortholog for this gene is PECT1, shown to be involved in respiration capacity in leaves (Otsuru *et al.* 2013). Associations with monthly precipitation overlapped less frequently than monthly temperature in adjacent months, likely due to the lower correlation of precipitation between adjacent months.

**Figure 3:**
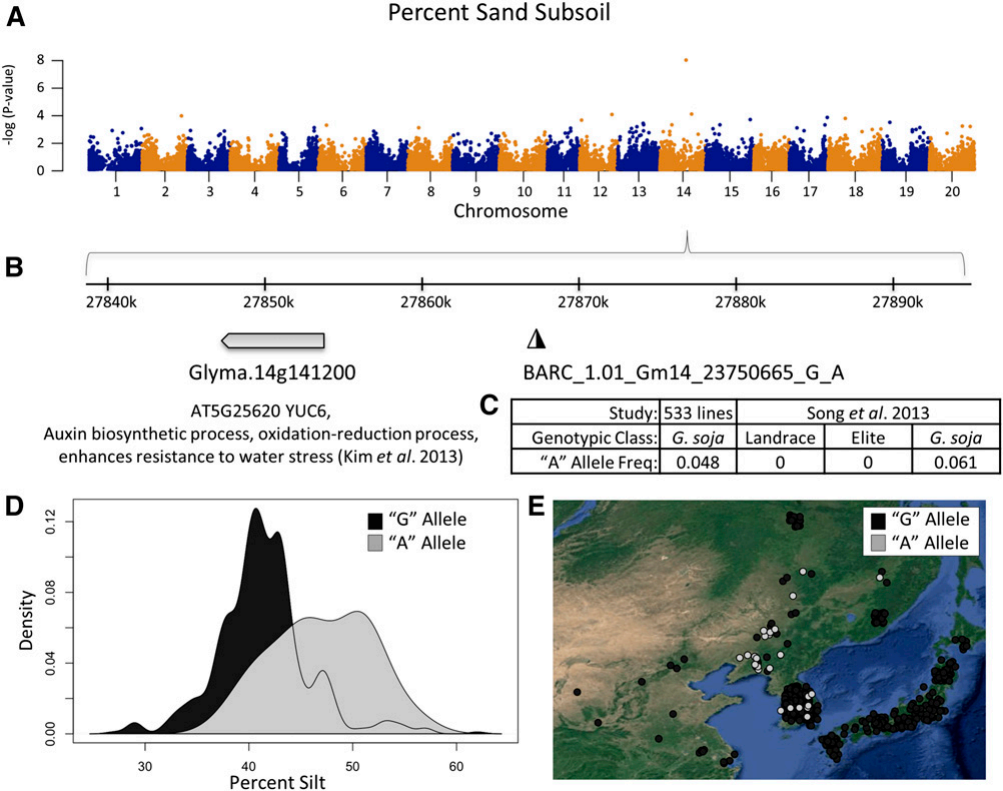
Genome-wide association results of percent sand, and percent silt. (A) Genome-wide view of association results for Percent Sand Subsoil. (B) Zoom in on 60 kb region around the significant marker BARC_1.01_Gm14_23750665_G_A, the most significant hit for topsoil and subsoil percent sand, and topsoil and subsoil percent silt. The “A” allele at this locus is associated with high silt environments, and is not found in a previous scan of landrace and elite soybean cultivars. The Arabidopsis ortholog for the nearest gene, Glyma.14g141200, is YUC6, a gene associated with enhanced resistance to water stress. (C) The “A” allele is rare in our sample, and found to be rare, or not present, in a previous screen of soybean genotypic classes (Song *et al.* 2013). (D) Density plot of allele frequency distribution for Percent Silt. The individuals with the “G” allele are shaded in dark gray overlaid with the “A” allele individuals in light gray. (E) Geographic location of individuals with the “G” allele (dark gray), or “A” allele (light gray), with jitter added to show overlapping samples. Individuals with missing genotyping data at this SNP are not shown.

The strongest association with a biophysical (soil) variable was an association between SNP ‘BARC_1.01_Gm14_23750665_G_A,’ on chromosome 14, and Percent Sand Subsoil (Figure 3A). This marker was also significant for Percent Sand Topsoil, Percent Silt Topsoil and Subsoil, and Cation Exchange Capacity Topsoil. As shown in Figure 3, this SNP occurs in a pericentromeric region with low SNP density in the SoySNP50K assay. The gene Glyma.14g141200 occurs in this region, and has the Arabidopsis ortholog YUC6 (Figure 3B), which is involved in the auxin biosynthesis pathway that provides enhanced resistance to water stress (Kim *et al.* 2013). The nonreference variant was rare in our sample, and not present in elite lines or landraces in a previous study (Figure 3C) (Song *et al.* 2013). Distributions of Topsoil Percent Silt and Percent Sand reveal that individuals carrying the nonreference variant were present in locations with 6% higher silt and 9% lower sand, on average, than individuals carrying the reference variant (Figure 3D and Figure S11). This is not merely a result of population structure, as the accessions with the nonreference variant are widely dispersed (Figure 3E).

For a complete list of significant markers see Table S7, or Figure S12 for Manhattan plots of the mixed model association results for each of the bioclimatic and biophysical variables.

### F_ST_ and SPA outliers

**Figure 4:**
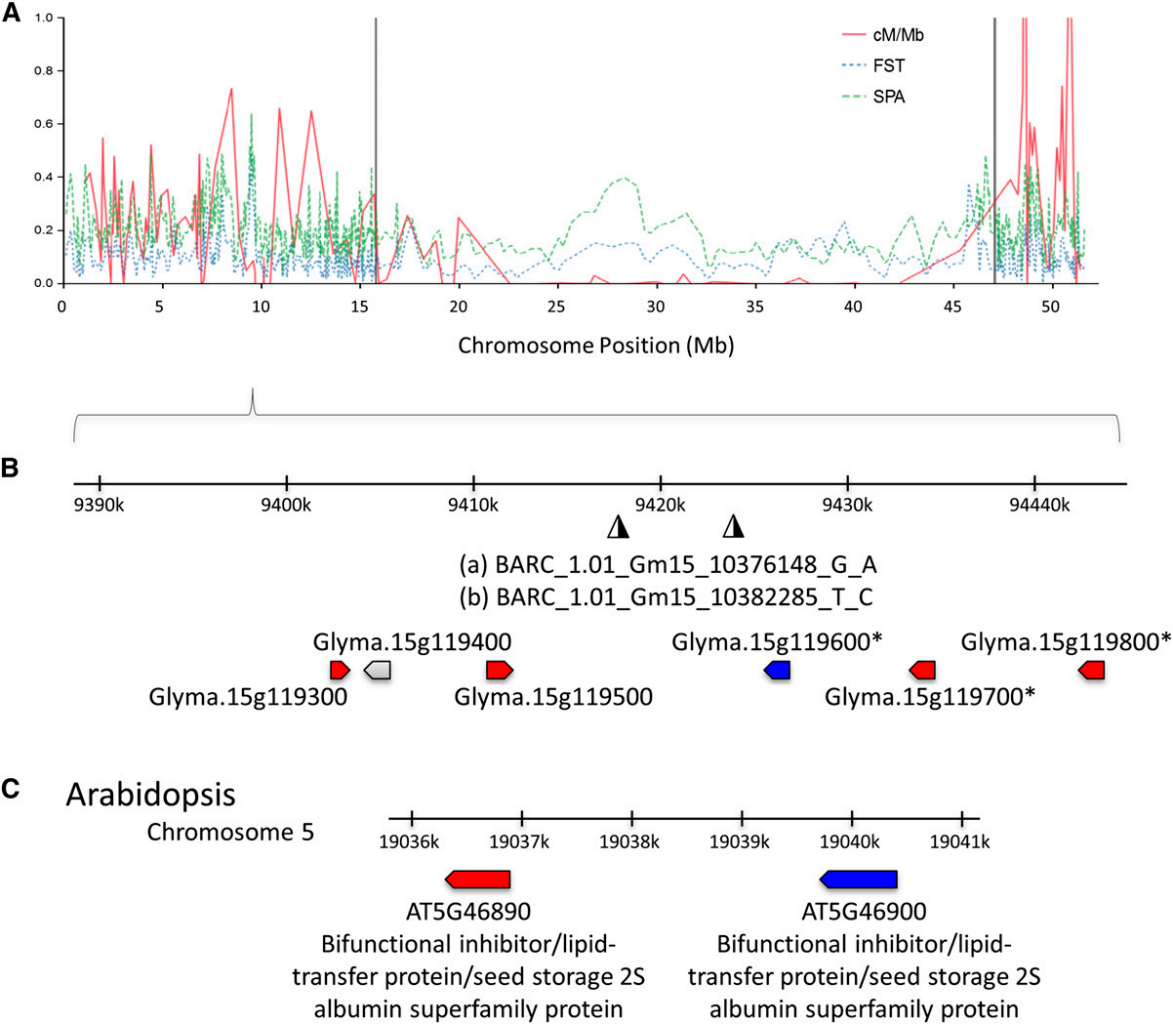
SPA, *F*_ST_, and recombination rate in the *G. soja* genome. (A) Sliding window of these values plotted on chromosome 15. Recombination decreases dramatically through the pericentromeric region, denoted by the vertical gray dotted lines. (B) Zoom in on 60 kb region around two significant SPA markers BARC_1.01_Gm15_10376148_G_A, and BARC_1.01_Gm15_10382285_T_C, a region of notably low recombination, and both high *F*_ST_ and SPA values. Three genes in this region (denoted with asterisks) were previously found to be duplicated, or deleted, in some elite soybean lines (Anderson *et al.* 2014). This cluster of genes appear to be members of a gene family. The Arabidopsis ortholog for the genes denoted in red is AT5G46890, a bifunctional inhibitor/lipid-transfer protein/seed storage 2S albumin superfamily protein. Similarly, the Arabidopsis ortholog for Glyma.15g119600, denoted in blue, is AT5G46900, a bifunctional inhibitor/lipid-transfer protein/seed storage 2S albumin superfamily protein. The implications of structural variation relating to *F*_ST_, SPA hits, or recombination are not yet clear.

SPA and *F_ST_* outlier analyses were used to identify allele frequencies that could indicate the action of selection at a locus. SPA outliers tended to divide the sample along the same axes identified in genetic assignment and PCA analyses. SPA selection scores were positively correlated with *F_ST_* (*r* = 0.76, *r*^2^ = 0.58; Figure S13 and Figure S14). The 99.9% outliers from SPA (Table S8) and *F_ST_* (Table S9) overlapped at two SNPs (9% of outliers) (Figure 4A). One of these overlapping SNPs had the highest SPA selection score. These two outlier SNPs occur in a gene family cluster on chromosome 15 (Figure 4B) with Arabidopsis orthologs annotated as, “bifunctional inhibitor/lipid-transfer protein/seed storage 2S albumin superfamily protein” (Figure 4C). SPA outliers were significantly enriched for genic space, whereas *F*_ST_ outliers and significant environmental associations were enriched for nongenic space (Table S10 and Figure S15).

## Discussion

### Population structure of Glycine soja

Genetic assignment analysis of *G. soja* in East Asia identified three primary clusters: Mainland North, Mainland South, and Island. The Mantel test identified isolation by distance, a result consistent with previous observations of differentiation in *G. soja* based on a latitude gradient and flowering time (Cregan and Hartwig 1984; Nakayama and Yamaguchi 2002). The Sea of Japan likely contributed as a physical barrier to migration between the Island cluster and the mainland clusters. All clusters had relatively small private allele richness compared to other plant species (Fang *et al.* 2014; Cornille *et al.* 2015). The Mainland North cluster had a pattern of elevated *F*_ST_, and the smallest number of polymorphic SNPs. The allele frequency differences in this cluster could be attributable to small local effective population size, and a higher level of genetic drift. Ascertainment bias likely also contributed to this pattern, as the Mainland North cluster was not represented in the discovery panel. The SoySNP50K discovery panel included two *G. soja* accessions: PI468916 (Mainland South), and PI479752 (collected in China but does not have longitude and latitude) (Song *et al.* 2013).

We observed a pattern of elevated *F*_ST_ in pericentromeric regions (Figure 4). Reduced recombination rates, as observed in pericentromeric regions of *G. max* (Schmutz *et al.* 2010), can contribute to elevated allele frequency divergence. This effect has been attributed to linked selection (Charlesworth *et al.* 1993).

### Agronomic implications of environmental association

The identification of genetic variants associated with higher temperatures or lower moisture could contribute to an understanding of the genetic basis of plant response to climate change. This, in turn, could enable breeders to identify individual accessions of a crop wild relative that may have alleles associated with abiotic stress tolerance. The nonreference variant detected in association with “Mean Temperature Wettest Quarter” on chromosome 8, which is associated with higher temperature, is found in ∼90% of our *G. soja* samples, while a previous study found this variant in 3.6% of elite soybean lines (Song *et al.* 2013). Another locus with potential effect on temperature response was detected on chromosome 16, this variant was present in accessions sampled from the Korean Peninsula north into Russia, corresponding to cooler growing season temperatures. The variant on chromosome 16 occurs at a moderate frequency (30%) in elite lines. These findings suggest that naturally occurring variants at these loci could contribute to improved drought response in elite soybeans.

The use of soils data allows us to examine how genetic variation may associate with below-ground environmental variation. While most of the root mass in soybeans occurs in the topsoil, the same SNPs were often associated with topsoil and subsoil variables. Soil texture and content associations identified several associations that have potential agronomic applications. For example, SNP ‘BARC_1.01_Gm04_3461538_T_C’, on chromosome 4 was associated with “soil pH” (Figure S16), which may be relevant in the response to iron deficiency chlorosis (IDC). IDC is not necessarily a shortage of iron in the soil, but rather the inability of the plant to uptake iron under certain conditions (Hansen *et al.* 2003). IDC has a number of soil and environmental factors associated with its severity, including high early season moisture, low temperature, and high soil pH (Hansen *et al.* 2003). The gene closest to the variant (3 kb away) is Glyma.04g044000, whose reported Arabidopsis ortholog is NRAMP2, known to be essential for Arabidopsis seed germination, and development in low iron conditions (Lanquar *et al.* 2005).

### Utility of complementary approaches

The SPA and *F*_ST_ outlier estimates identified no overlap with the set of SNPs identified in the environmental association. SPA and *F*_ST_ identify SNPs on the basis of strong allele frequency differentiation, under the assumption that the difference is due to differential selection pressures. Environmental association explicitly tests covariation between allele frequency and a bioclimatic or biophysical variable. Since we are taking an outlier approach, the minimal overlap between these approaches can be attributed to differing assumptions of each test (reviewed in Akey 2009).

Copy number variants reported by Anderson *et al.* (2014), occur at the same genomic location as an allele frequency outlier in both the *F*_ST_ and SPA analyses. Recently, understanding the link between phenotypic variation and structural variation has been a topic of intense study: how do deletions or duplications ranging in size from single genes to sizeable pieces of chromosomes affect phenotypes (reviewed in Saxena *et al.* 2014)? It should be noted that we were unable to investigate copy number variation in *G. soja* with this dataset as we were querying single SNPs, which are not diagnostic of chromosomal structural variation, so the broader implications of the cooccurrence is unclear.

### Utility to plant breeding

These findings in *G. soja* could be especially beneficial for plant breeders focusing on abiotic stress tolerance. Compared to other staple crops, soybean improvement has rarely employed the genetic potential found in its crop wild relatives (Hajjar and Hodgkin 2007). This underutilization is likely related to the effort required to select lines from large germplasm collections, to make a multitude of crosses to create mapping populations, and to properly phenotype populations for specific traits. In soybean, there is also a limitation placed by the number of seeds produced from an individual cross. Environmental association is an attractive alternative, where the diversity in a germplasm collection is used to identify genetic differentiation owing to environmental or abiotic factors. We identify such promising loci (Table S7, Table S8, and Table S9), which could immediately be applied to a validation population, and implemented in a breeding program.

### Limitations and biases

While these results are promising, a number of limitations and biases with these data and approaches need to be considered. The limitations include the resolution of the bioclimatic and biophysical data, the assumptions made in treating environmental variables as a phenotype, and using an association mapping framework.

The data in the databases are limited to a specific resolution. That is, points a certain distance apart are indistinguishable by their bioclimatic or biophysical variables. This amounts to “phenotyping error” in our approach, and reduces the power of our tests. This is particularly true for biophysical (soil) variables, which may vary over much finer scales than the resolution of the database (Brady *et al.* 2005).

In treating the local environmental variables as a “phenotype,” our approach requires the assumption that these variables constitute the selective pressures on the populations. However, climatic variables are merely proxies for response to abiotic stresses, and we are assuming the individuals sampled are adapted to their local conditions. In a similar vein, our approach works best when a variant exhibits antagonistic pleiotropy, rather than conditional neutrality. If a variant is positively selected in one local environment, and negatively selected in another (antagonistic pleiotropy), our approach is more likely to identify it as significant than if it were neutral in the second environment (conditional neutrality) (Tiffin and Ross-Ibarra 2014).

Lastly, because we are taking an association mapping approach, the limitations of association analyses apply. The variants we identify are not assumed to be causative, but are presumed to be in LD with a causative variant. Limited marker density can increase the false negative rate as there is limited power to detect all but the strongest signals of selection (Tiffin and Ross-Ibarra 2014). Because we are using a fixed SNP platform, our results are affected by ascertainment bias. Since the SNPs are ascertained, we are biased toward common SNPs, and must make a common trait–common variant assumption. Our approach will miss rare alleles of large effect, or any structural variants that influence phenotypic variance. For more on the common variant–common trait assumption in association mapping, see Morrell *et al.* (2012).

### Conclusion

Data drawn from four large public databases (GRIN, WorldClim, ISRIC, and Soybase) were used to explore the intersection of bioclimatic, biophysical, and genetic components in the important soybean crop wild relative, *G. soja*. Genetic variation associated with the environmental variation across the native range of *G. soja* was identified. We focus on the identification of loci that could contribute to abiotic stress tolerance. Genome scans for environmental association provide an opportunity to categorize and evaluate the utility of genetic variants in germplasm collections, complimenting recent initiatives to categorize and evaluate international germplasm collections (http://www.divseek.org/, McCouch *et al.* 2013; Dempewolf *et al.* 2014). Ideally these results can play a role in improving crop tolerance to globally changing environmental conditions.

## Supplementary Material

Supporting Information
