# Supplementary material for: Environmental Association Analyses Identify Candidates for Abiotic Stress Tolerance in Glycine soja, the Wild Progenitor of Cultivated Soybeans
Source: G3 (Bethesda). 2016 Jan 27;6(4):835–43. doi: 10.1534/g3.116.026914 (PMC4825654; doi:10.1534/g3.116.026914)
Supplement: Supporting Information [file supp_6_4_835__index.html]

Environmental Association Analyses Identify Candidates for Abiotic Stress Tolerance in Glycine soja, the Wild Progenitor of Cultivated Soybeans — Supporting Information 

# Environmental Association Analyses Identify Candidates for Abiotic Stress Tolerance in *Glycine soja*, the Wild Progenitor of Cultivated Soybeans

## Supporting Information for Anderson *et al.*, 2016

**Files in this Data Supplement:**

- Figure S1 - Standardized distributions of biophysical (soil) and bioclimatic variables. (.tif, 1686 KB)
- Figure S2 - PC1 and PC2 of 533 individuals among *G. soja* accessions. (.tif, 976 KB)
- Figure S3 - Mixed-model association mapping results for Mean Temperature Wettest Quarter. (.tif, 2118 KB)
- Figure S4 - A) Density plots of pairwise similarity for each fastSTRUCTURE cluster of *G. soja* B) Folded site frequency spectrum of all markers in all individuals used in this study. (.tif, 784 KB)
- Figure S5 - LD decay in *G. soja*. (.tif, 648 KB)
- Figure S6 - Variation in ecogeographic variables across the range of *G. soja*. (.tif, 2546 KB)
- Figure S7 - Investigation of SNP: BARC\_1.01\_Gm08\_40882335\_A\_G distribution, the most significant marker associated with Mean Temperature Wettest Quarter. (.tif, 2152 KB)
- Figure S8 - One marker, BARC\_1.01\_Gm16\_1552499\_A\_G, was found significant in 11 temperature related bioclimatic variables. (.tif, 1649 KB)
- Figure S9 - Investigation of SNP: BARC\_1.01\_Gm16\_1552499\_A\_G distribution. (.tif, 1620 KB)
- Figure S10 - Genome-wide association significant marker for July Precipitation and Precipitation Wettest Quarter. (.tif, 2563 KB)
- Figure S11 - Investigation of SNP: BARC\_1.01\_Gm14\_23750665\_G\_A distribution. (.tif, 787 KB)
- Figure S12 - SoySNP50K Bioclimatic and Biophysical association results displayed in Manhattan plots. (.docx, 2853)
- Figure S13 - Genome-wide distribution of SPA scores (green dashed line), *F*ST (blue dotted line), and recombination rate (red solid line). (.tif, 2542 KB)
- Figure S14 - Correlation between SPA and *F*ST scores. (.tif, 1605 KB)
- Figure S15 - Enrichment analysis for genomic region. (.tif, 807 KB)
- Figure S16 - A) Genome-wide associations with topsoil pH and subsoil pH. B) Zoom in on 60 kb region around the significant marker BARC\_1.01\_Gm04\_3461538\_T\_C. (.tif, 1501 KB)
- Table S1 - PI Number, latitude, longitude, country, and STRUCTURE-identified population of origin for each of the accessions used in this study. (.txt, 22 KB)
- Table S2 - Translation table for position of the SoySNP50K SNPs on the Glyma v1 assembly and Glyma v2 assembly. (.txt, 3203 KB)
- Table S3 - Bioclimatic and Biophysical variables examined in this study. (.txt, 172 KB)
- Table S4 - Correlation between biophysical and bioclimatic variables, blue indicates a high positive correlation, white indicates a correlation near zero, and red indicates a high negative correlation. (.xlsx, 102 KB)
- Table S5 - Environmental association resulting p-value for each SNP and each environmental variable. (.zip, 13449 KB)
- Table S6 - Environmental association resulting FDR-value for each SNP and each environmental variable. (.zip, 11742 KB)
- Table S7 - Significant hits from environmental association results. (.xlsx, 57 KB)
- Table S8 - Significant markers from SPA analysis. (.xlsx, 15 KB)
- Table S9 - SNP name, *G. soja*, closest annotated gene model and Arabidopsis thaliana homologue for the top 0.1% of FST outliers. (.xlsx, 12 KB)
- Table S10 - Genomic location enrichment analysis, bold text indicates a significant enrichment. (.xlsx, 11 KB)
