# Supplementary figures and images for: Environmental Association Analyses Identify Candidates for Abiotic Stress Tolerance in Glycine soja, the Wild Progenitor of Cultivated Soybeans
Source: G3 (Bethesda). 2016 Jan 27;6(4):835–43. doi: 10.1534/g3.116.026914 (PMC4825654; doi:10.1534/g3.116.026914)

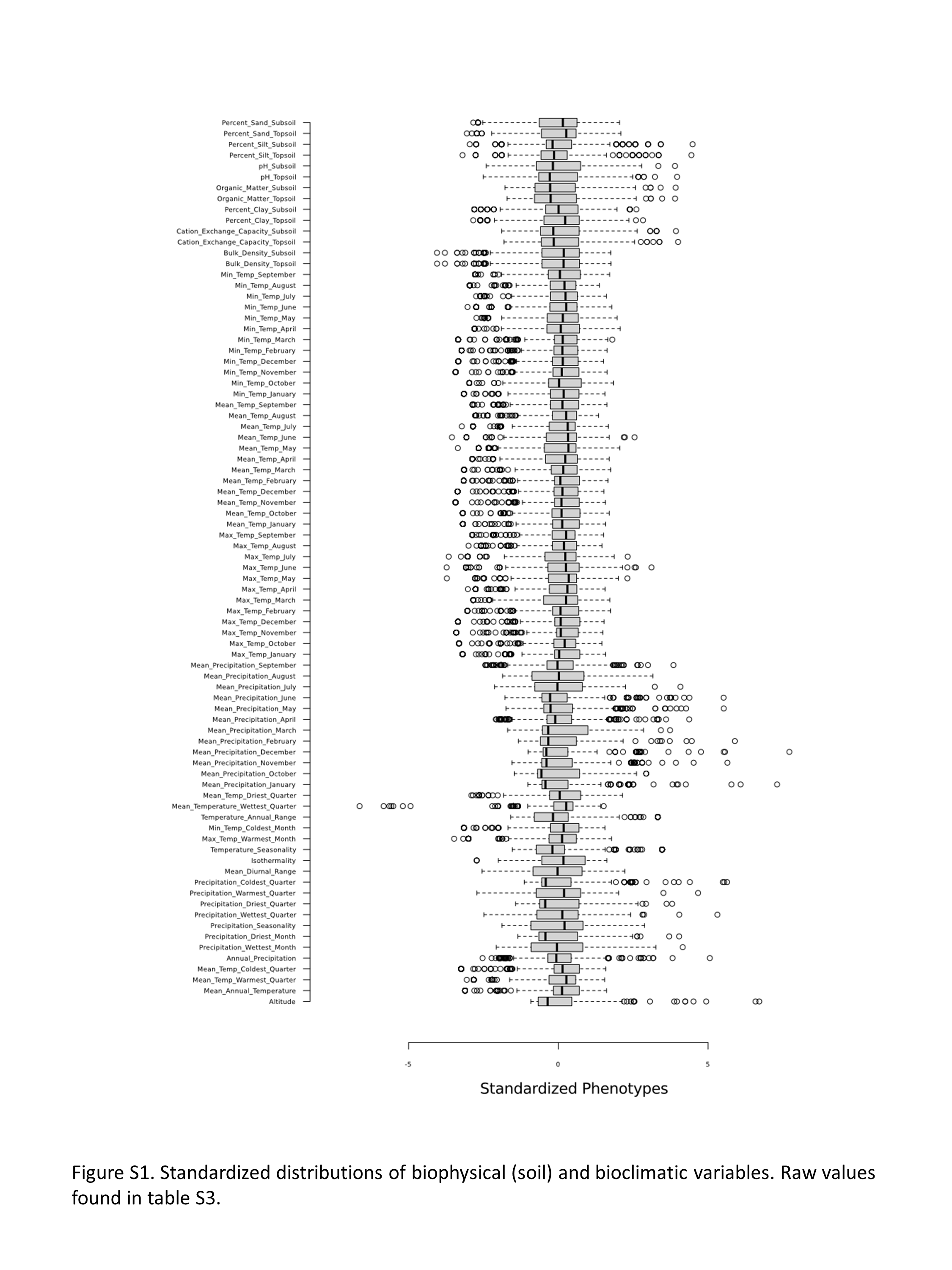

Supplement: Supporting Information [file supp_g3.116.026914_FigureS1.tif]

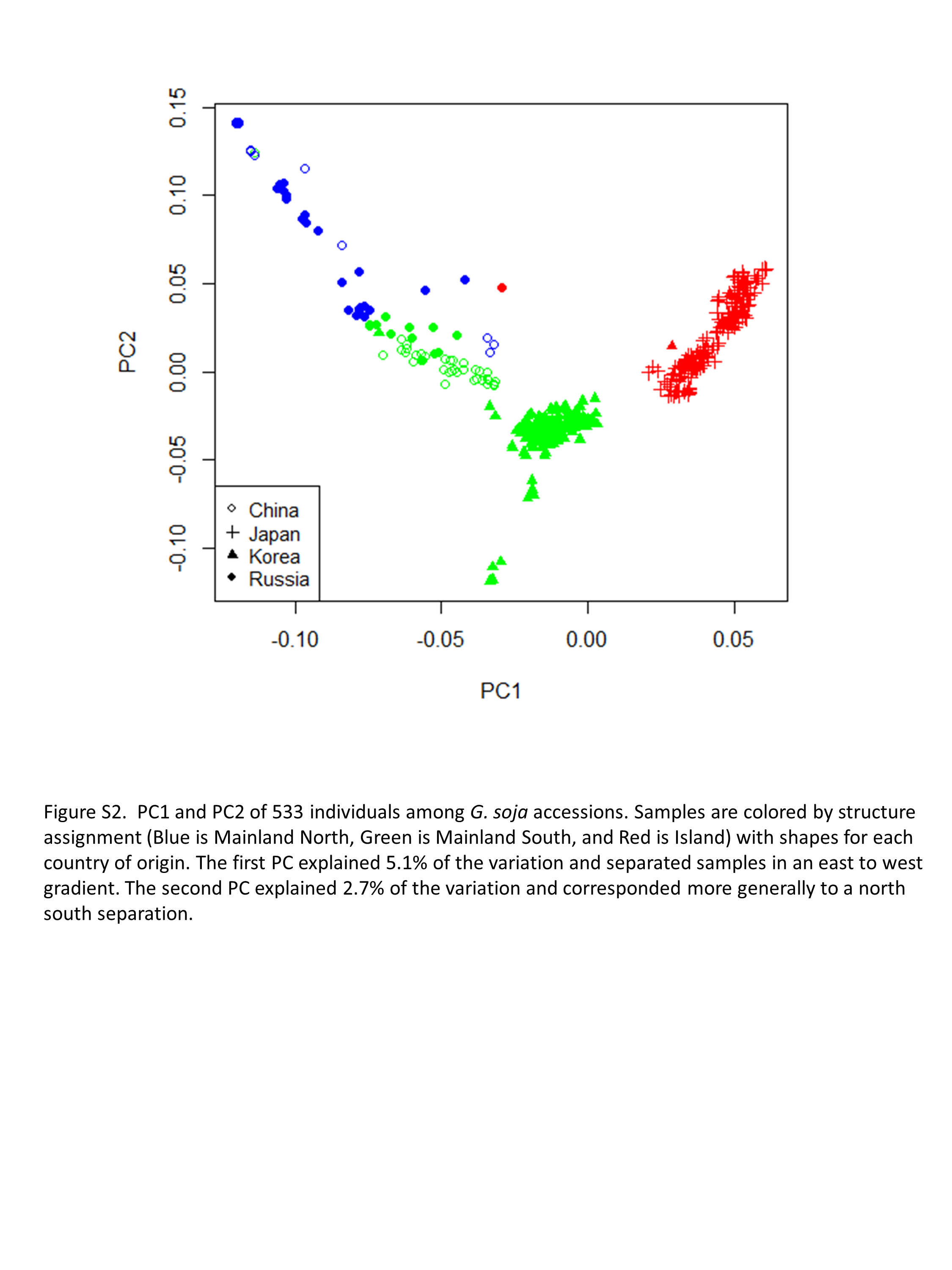

Supplement: Supporting Information [file supp_g3.116.026914_FigureS2.tif]

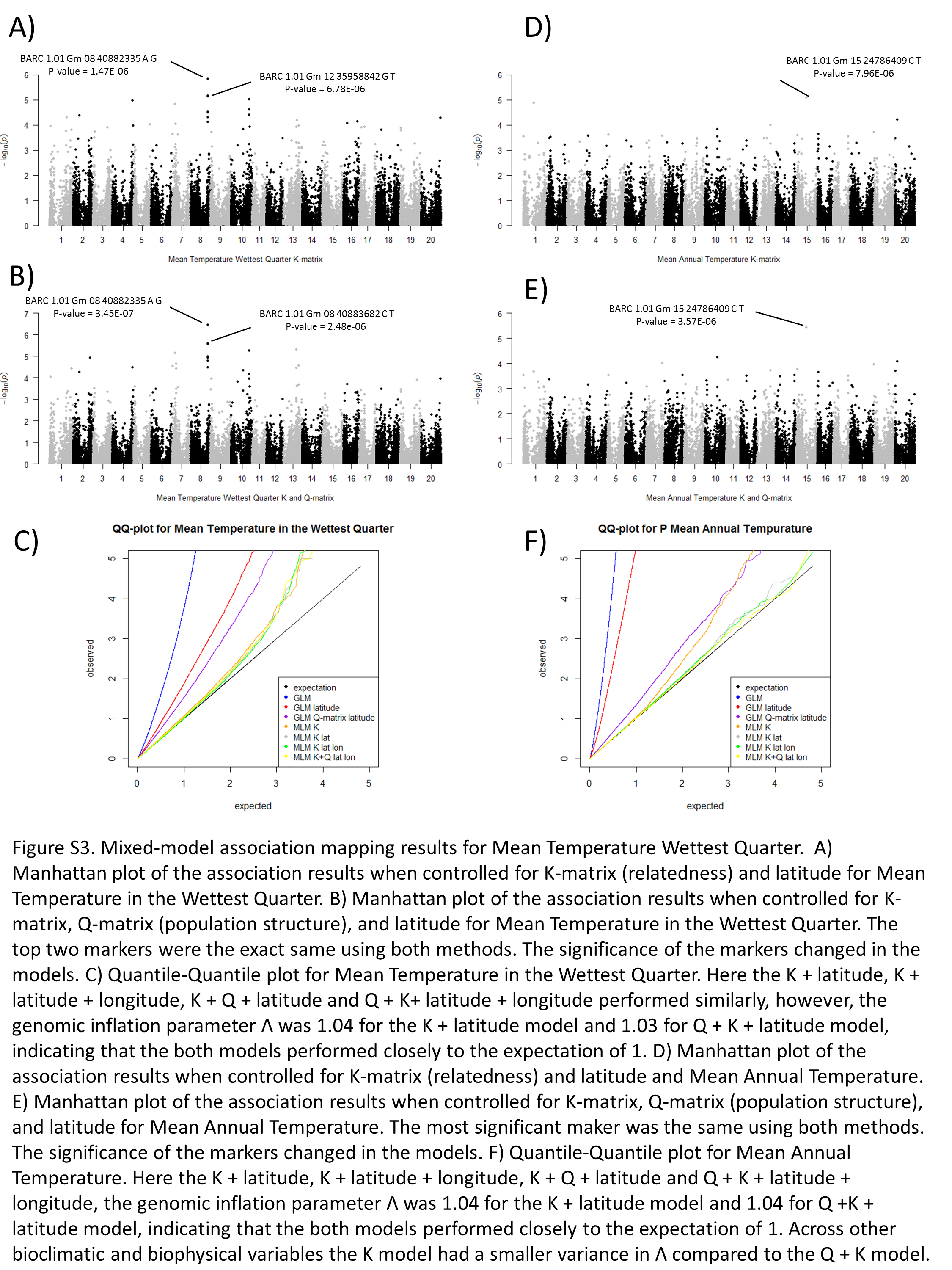

Supplement: Supporting Information [file supp_g3.116.026914_FigureS3.tif]

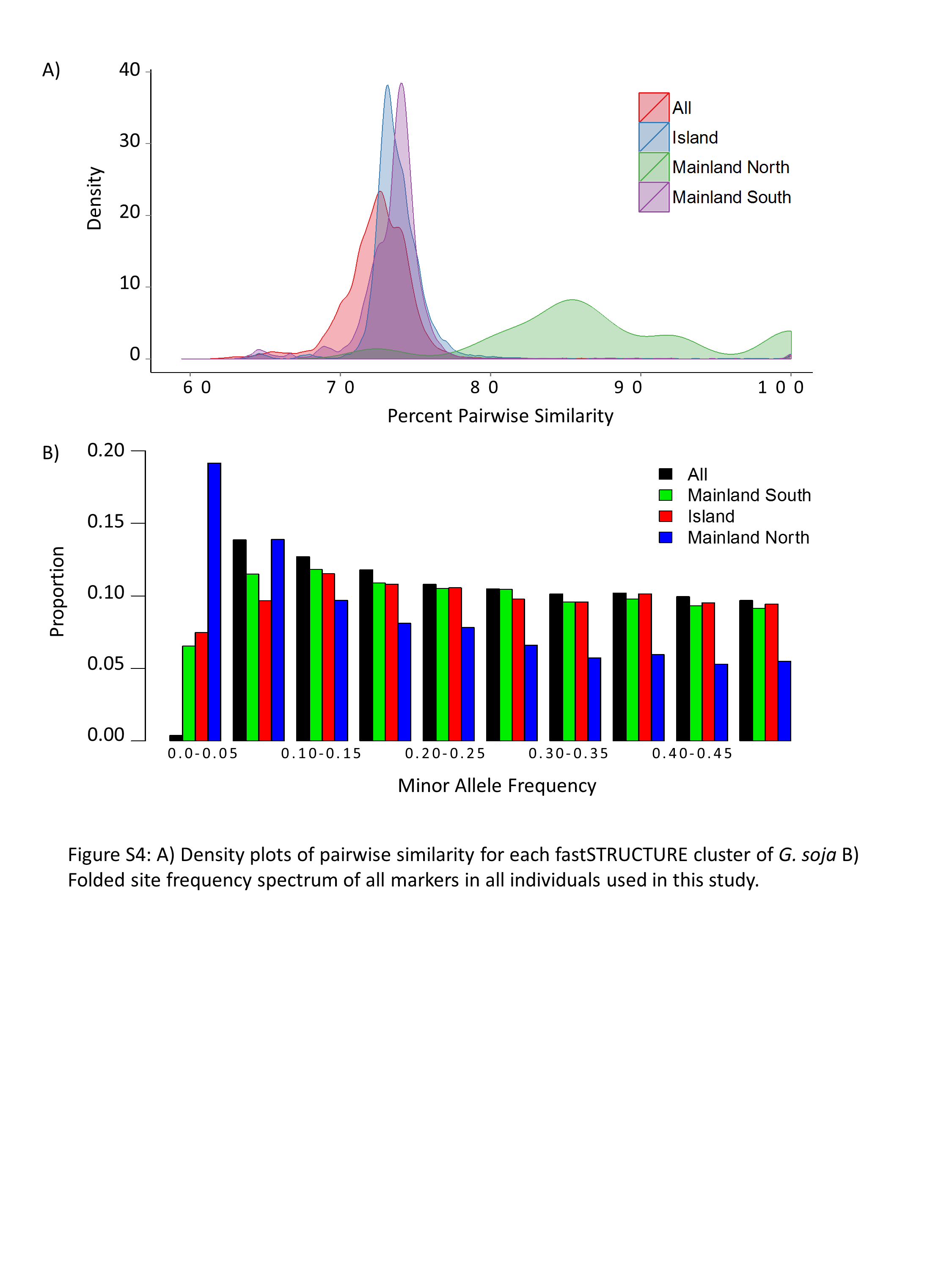

Supplement: Supporting Information [file supp_g3.116.026914_FigureS4.tif]

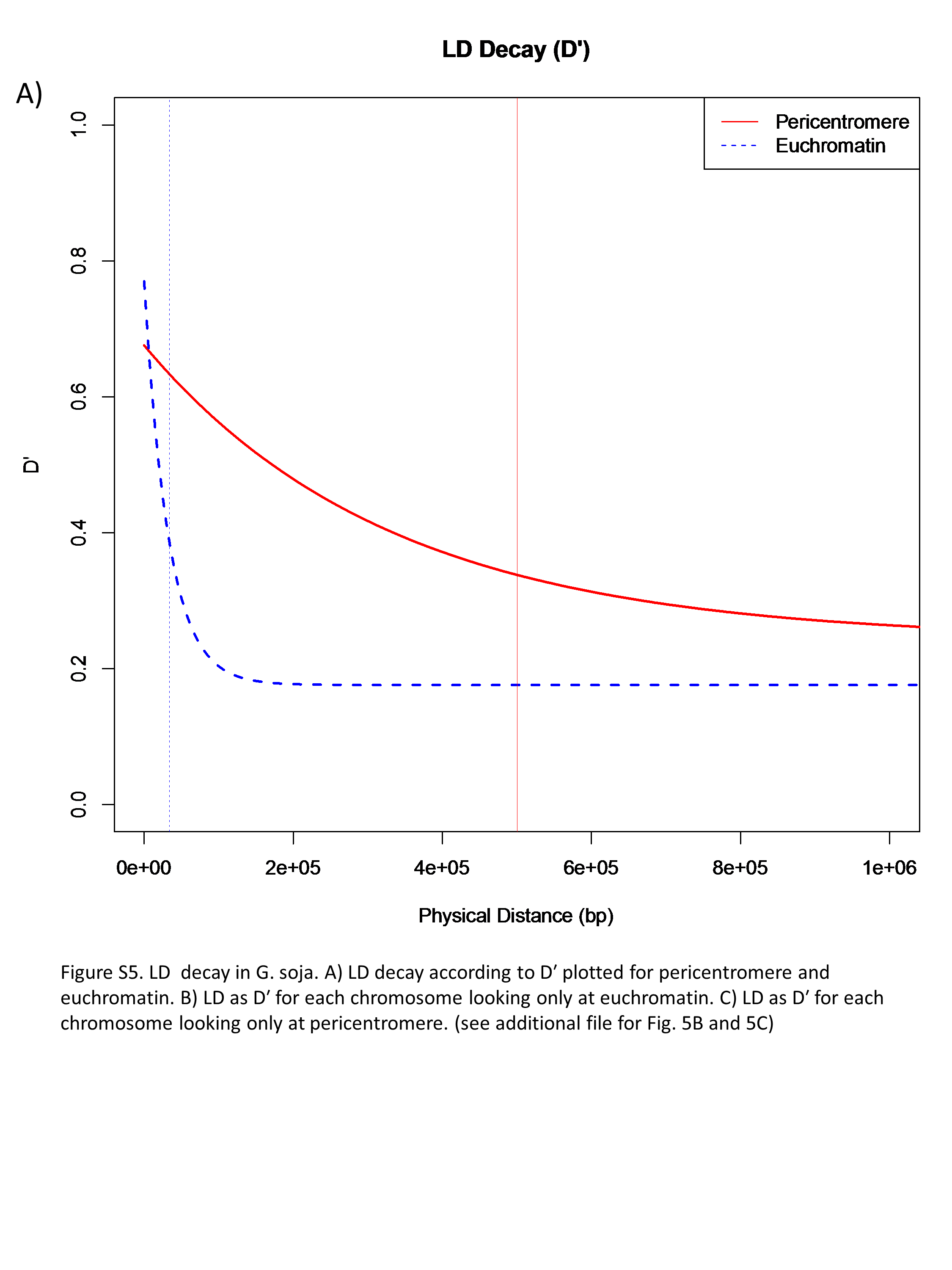

Supplement: Supporting Information [file supp_g3.116.026914_FigureS5.tif]

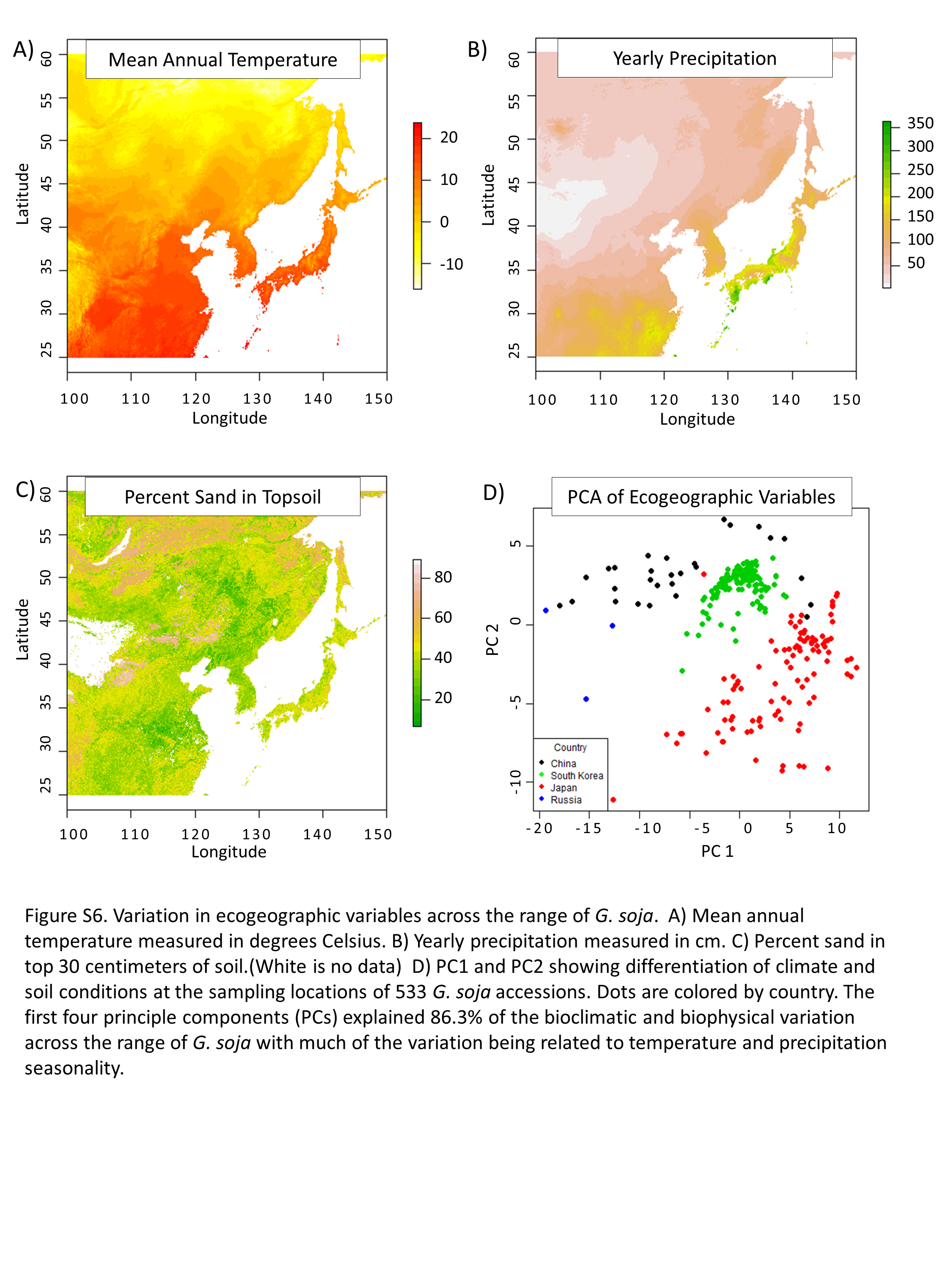

Supplement: Supporting Information [file supp_g3.116.026914_FigureS6.tif]

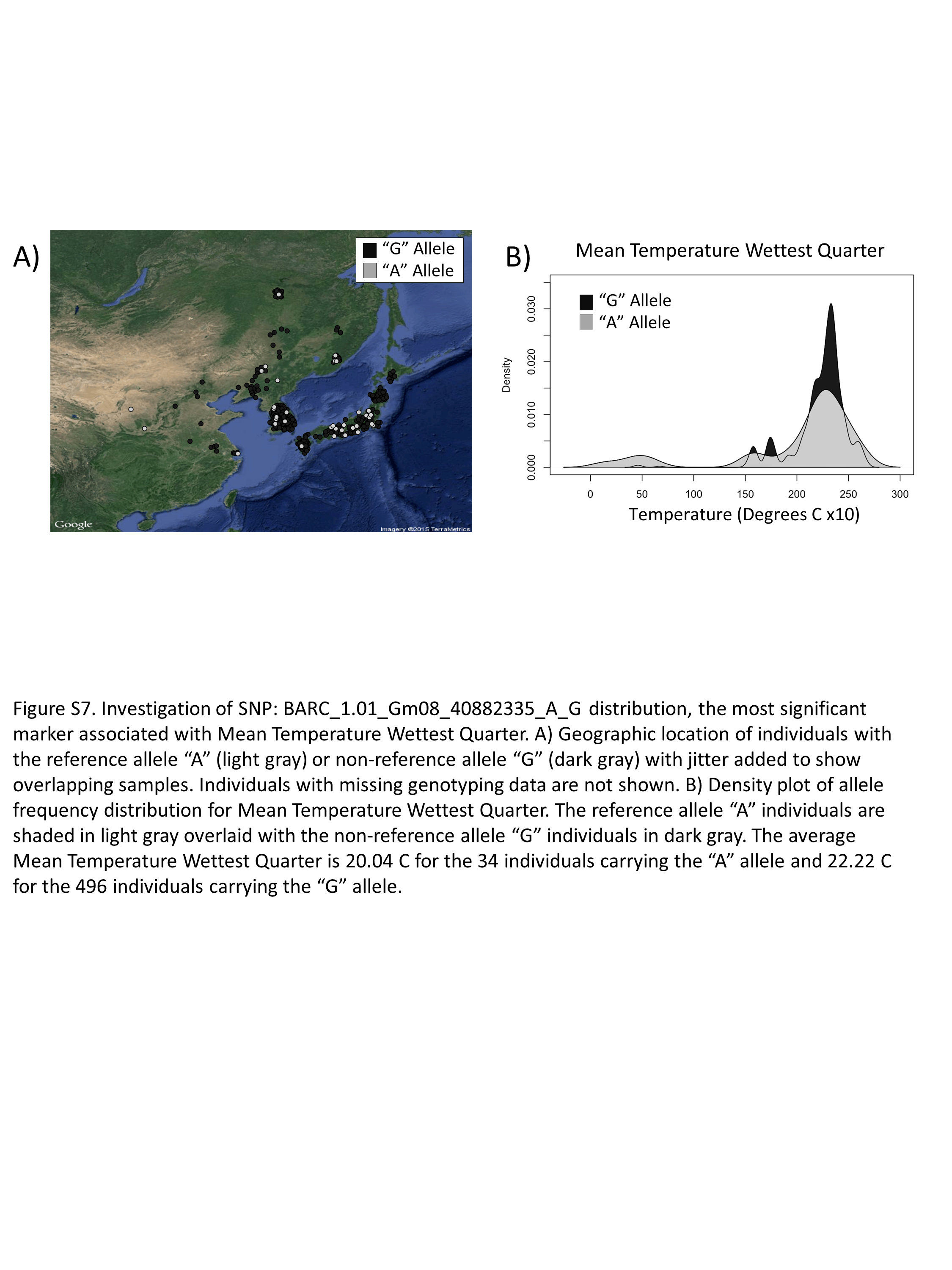

Supplement: Supporting Information [file supp_g3.116.026914_FigureS7.tif]

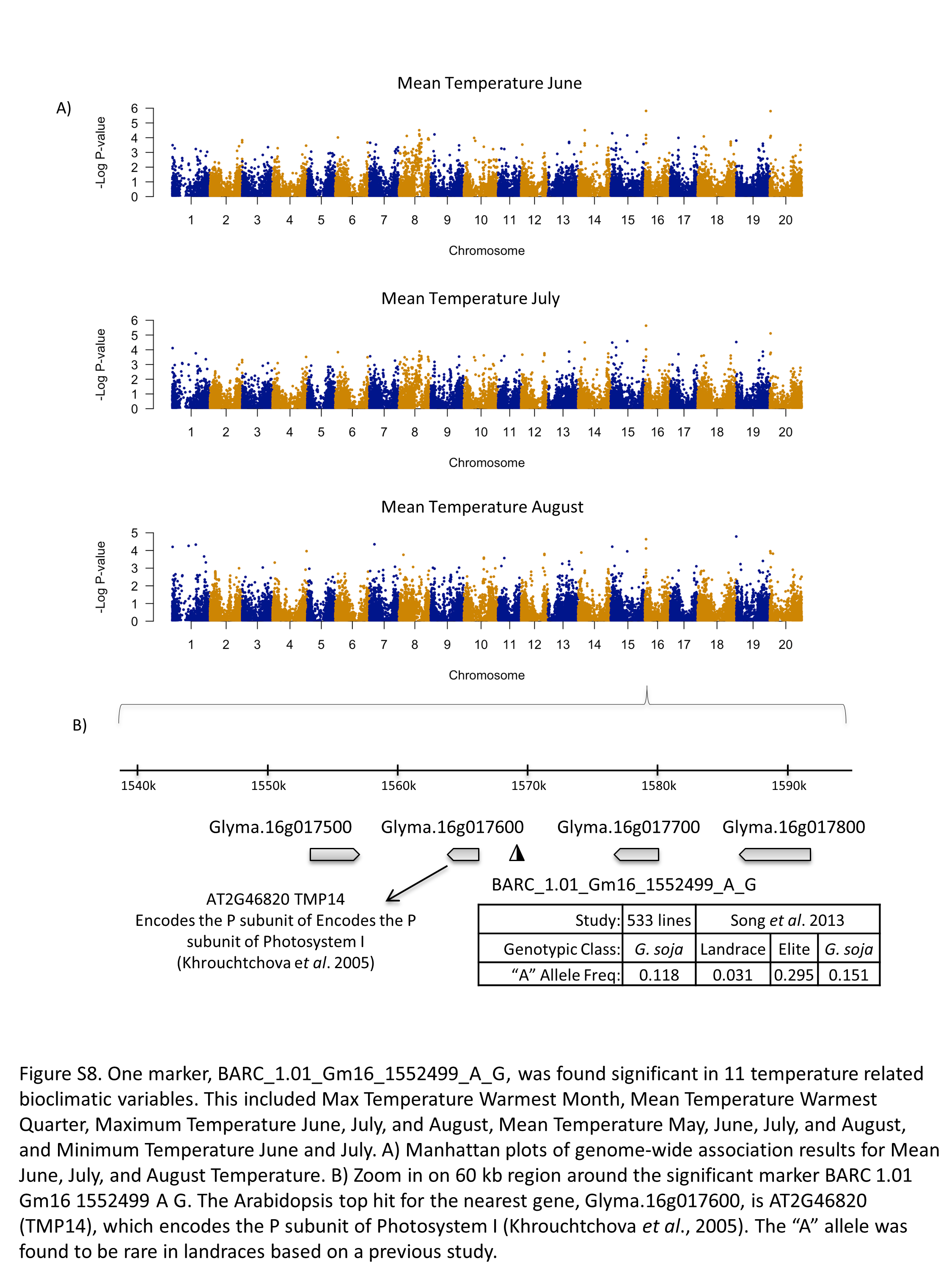

Supplement: Supporting Information [file supp_g3.116.026914_FigureS8.tif]

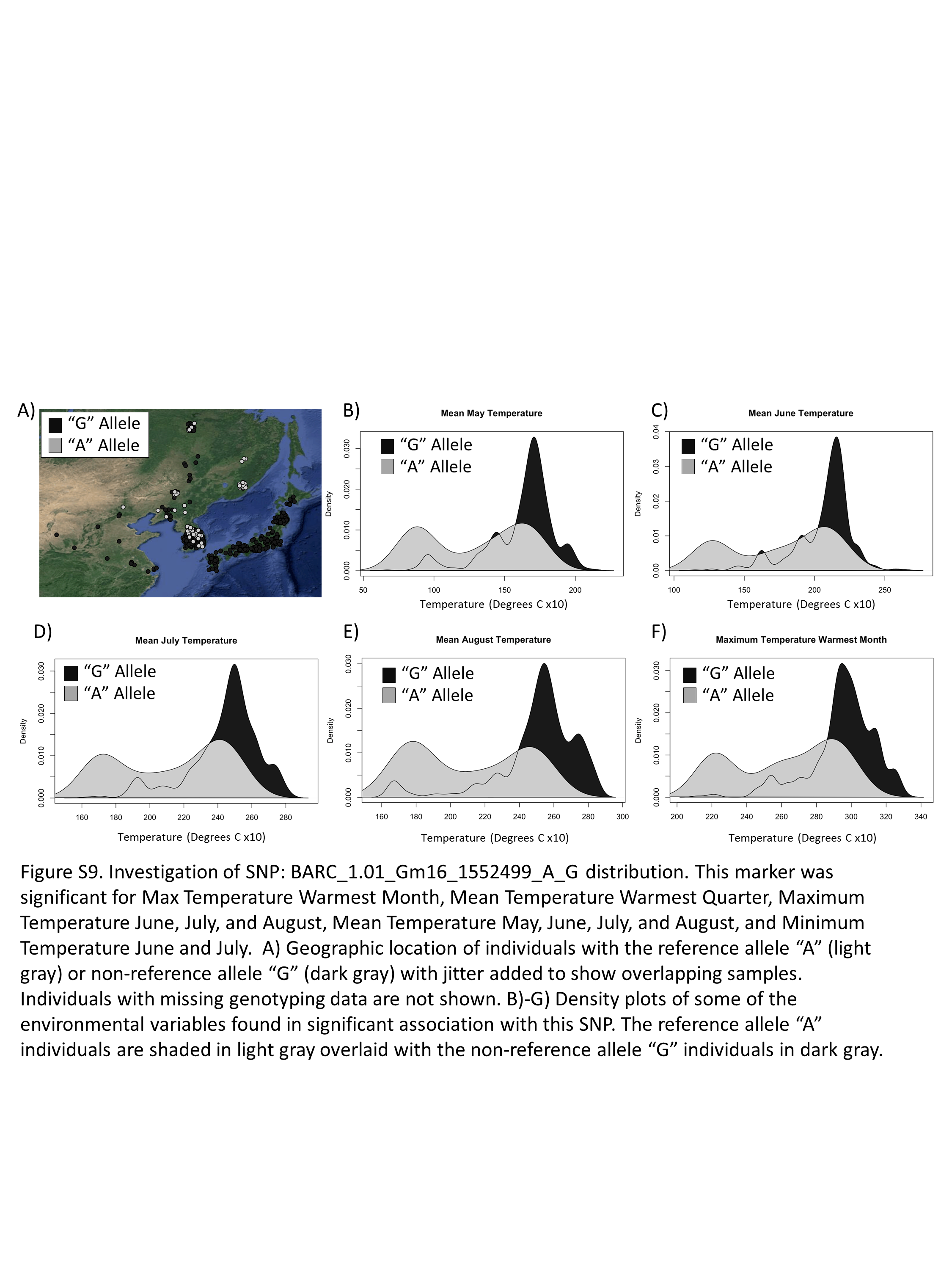

Supplement: Supporting Information [file supp_g3.116.026914_FigureS9.tif]

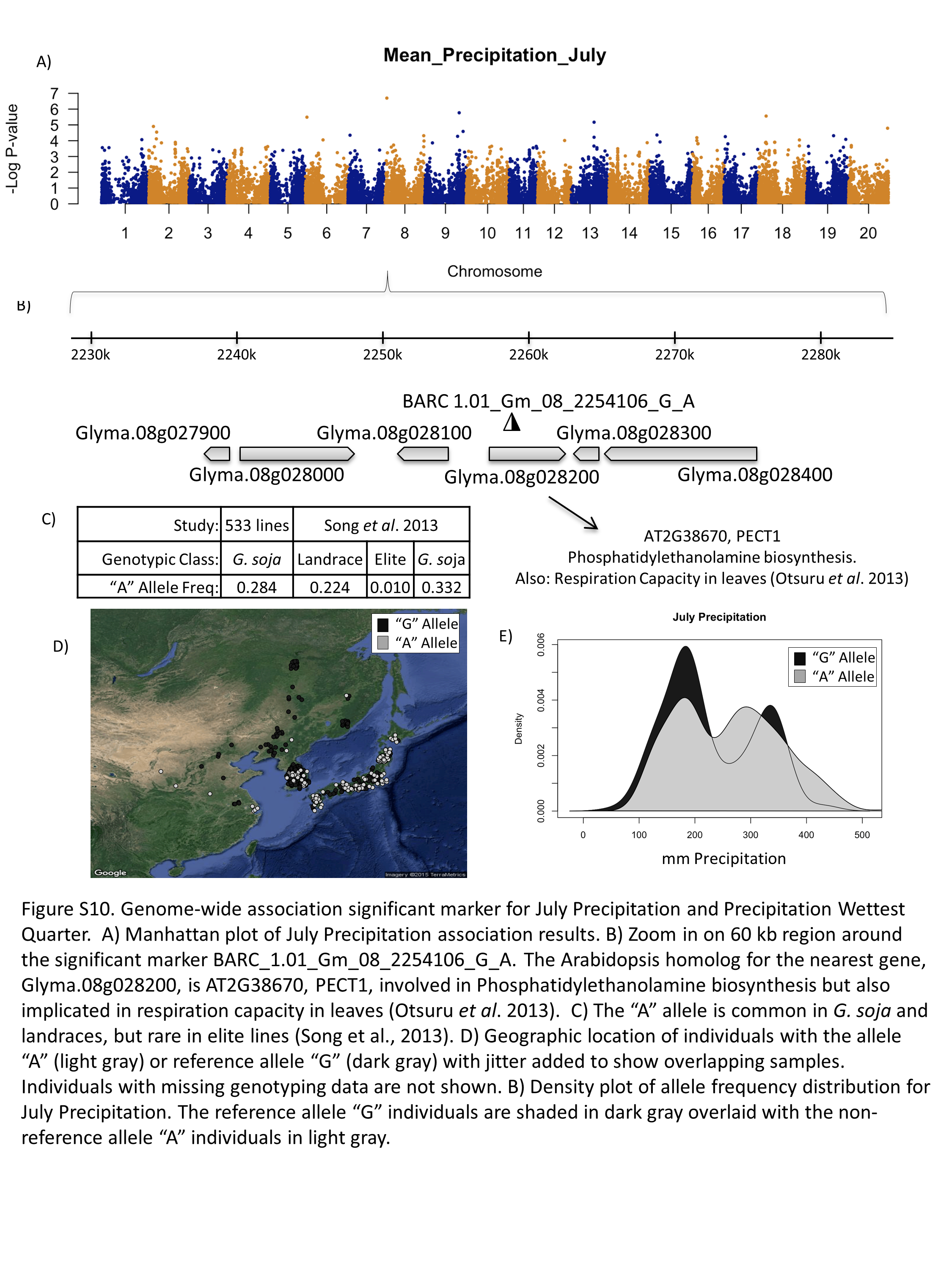

Supplement: Supporting Information [file supp_g3.116.026914_FigureS10.tif]

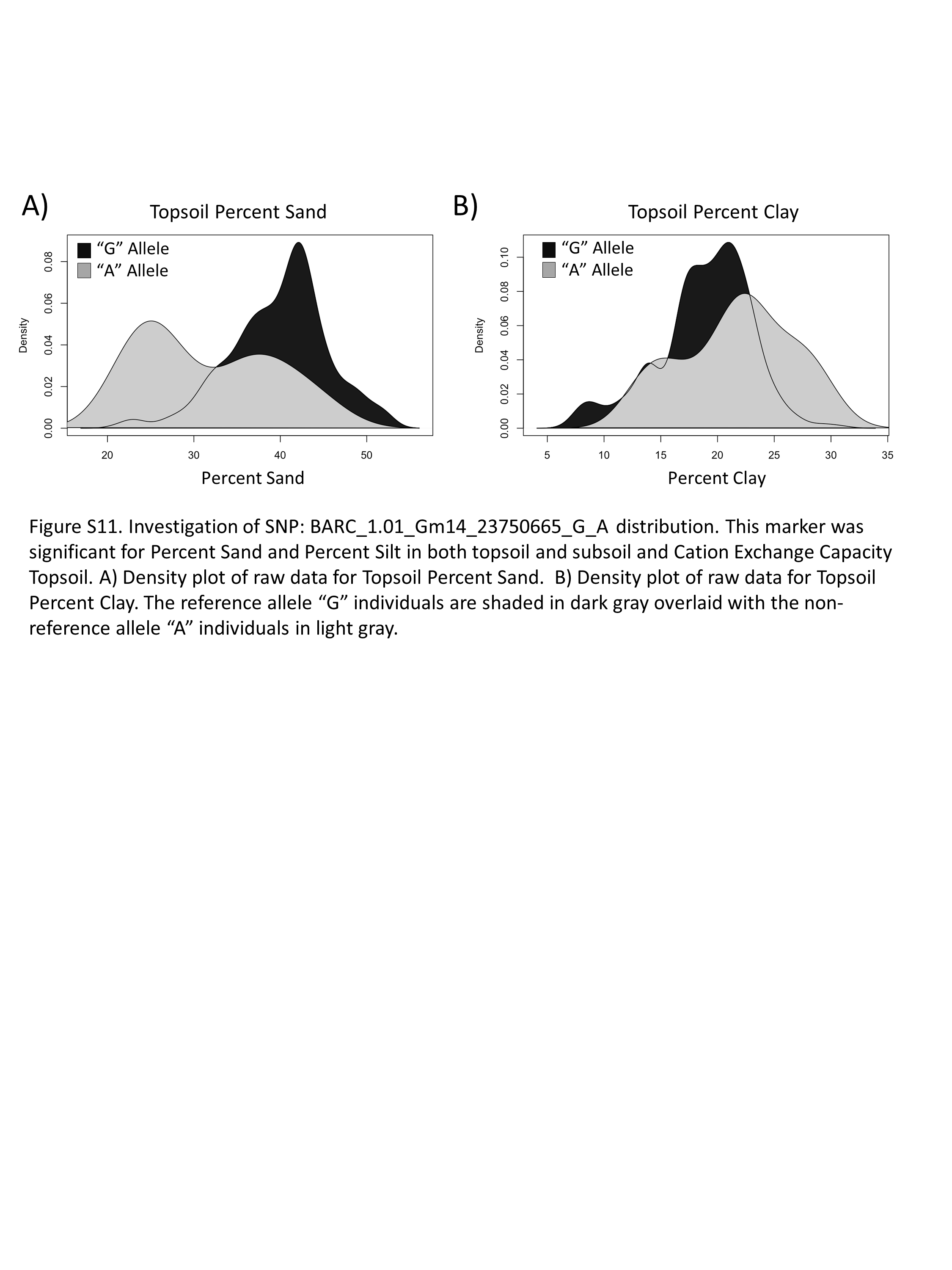

Supplement: Supporting Information [file supp_g3.116.026914_FigureS11.tif]

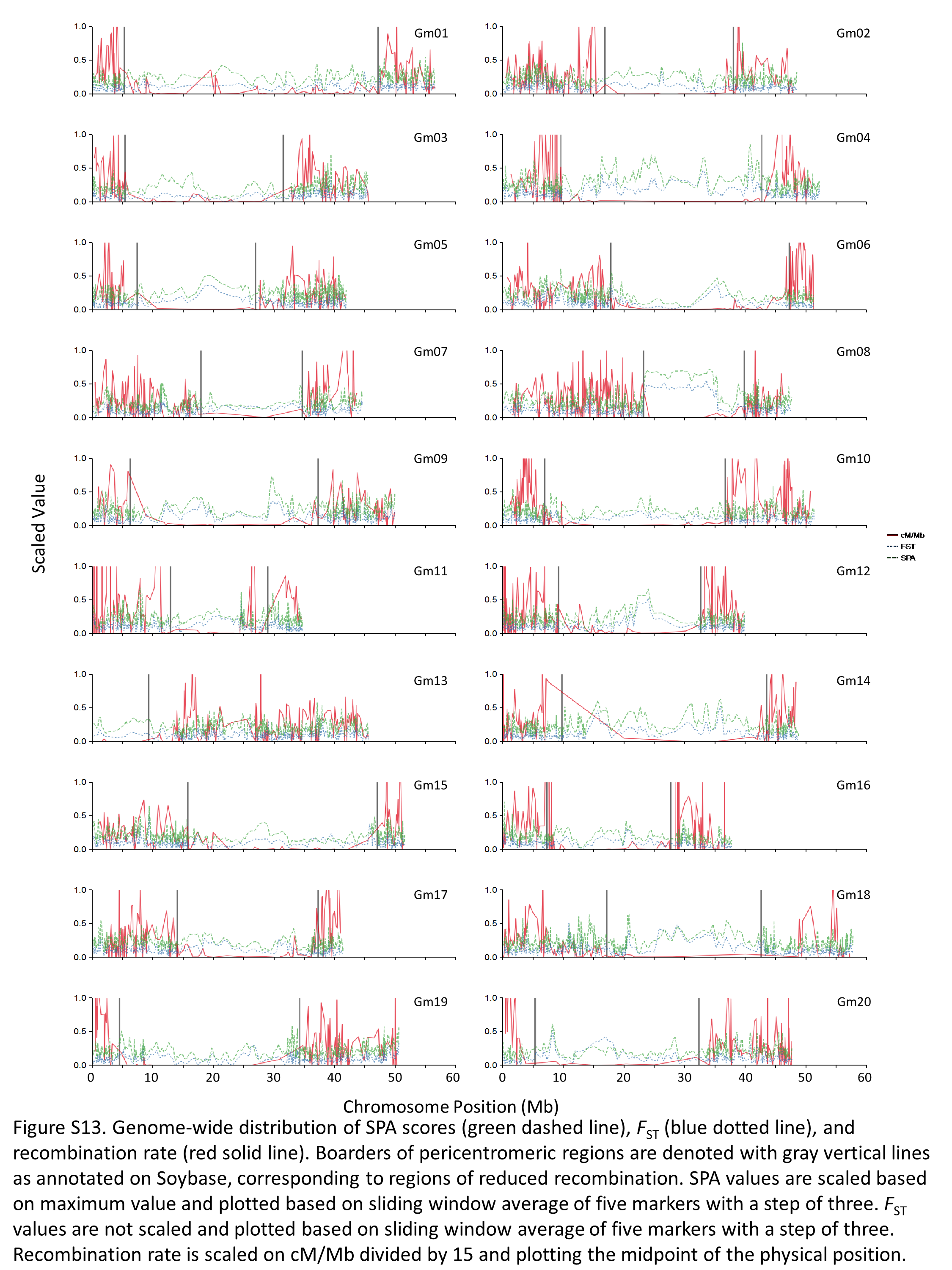

Supplement: Supporting Information [file supp_g3.116.026914_FigureS13.tif]

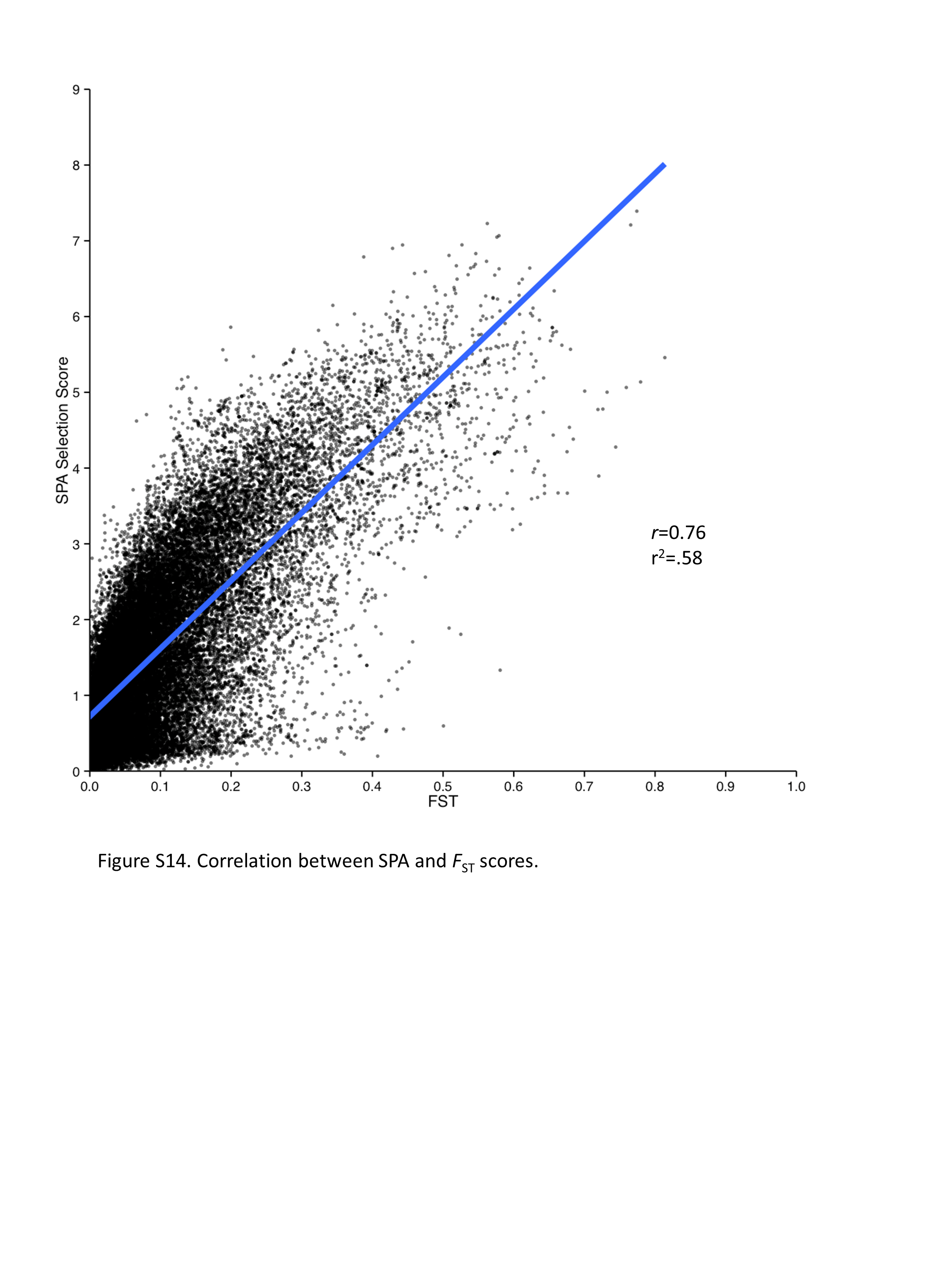

Supplement: Supporting Information [file supp_g3.116.026914_FigureS14.tif]

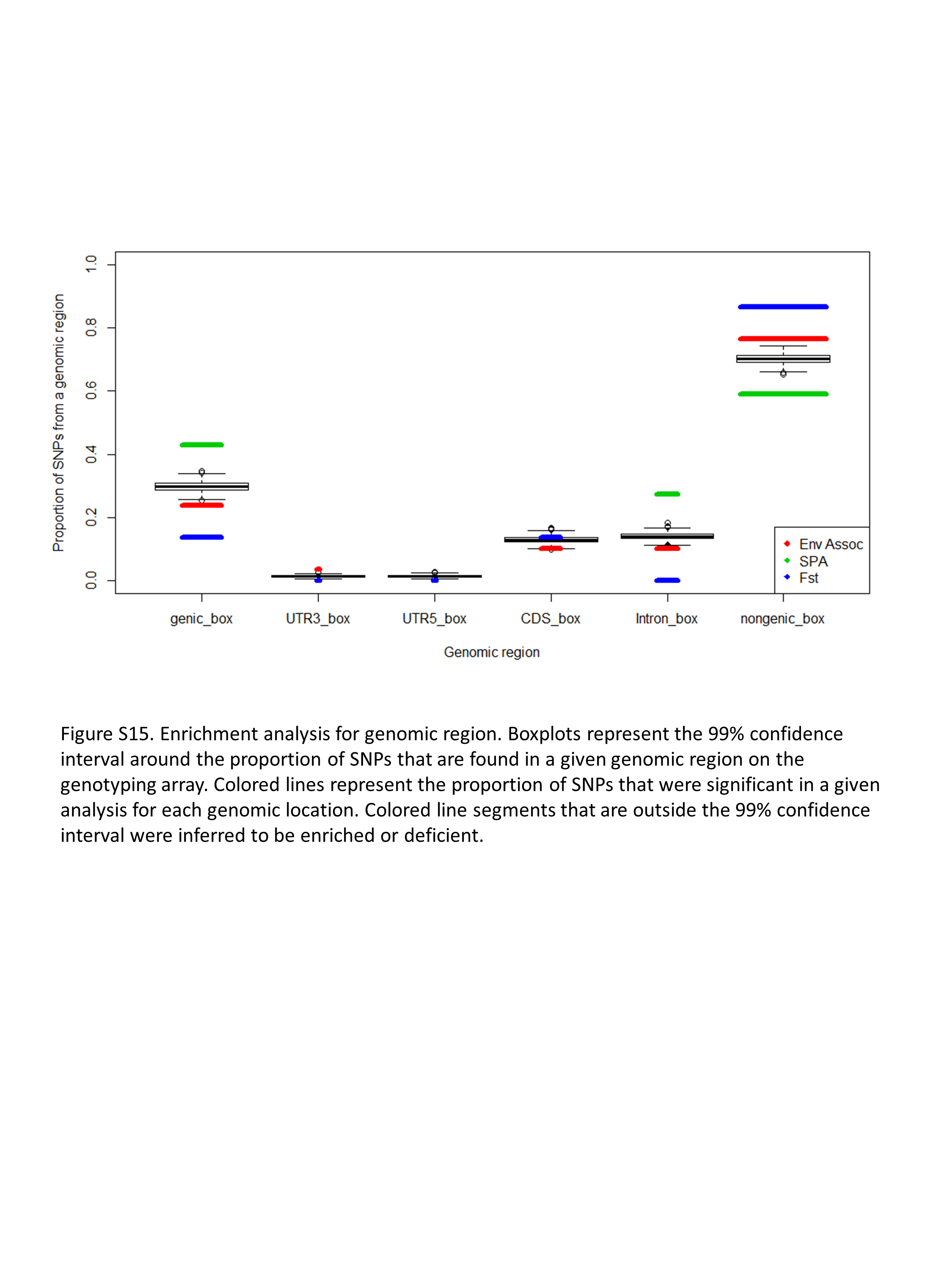

Supplement: Supporting Information [file supp_g3.116.026914_FigureS15.tif]

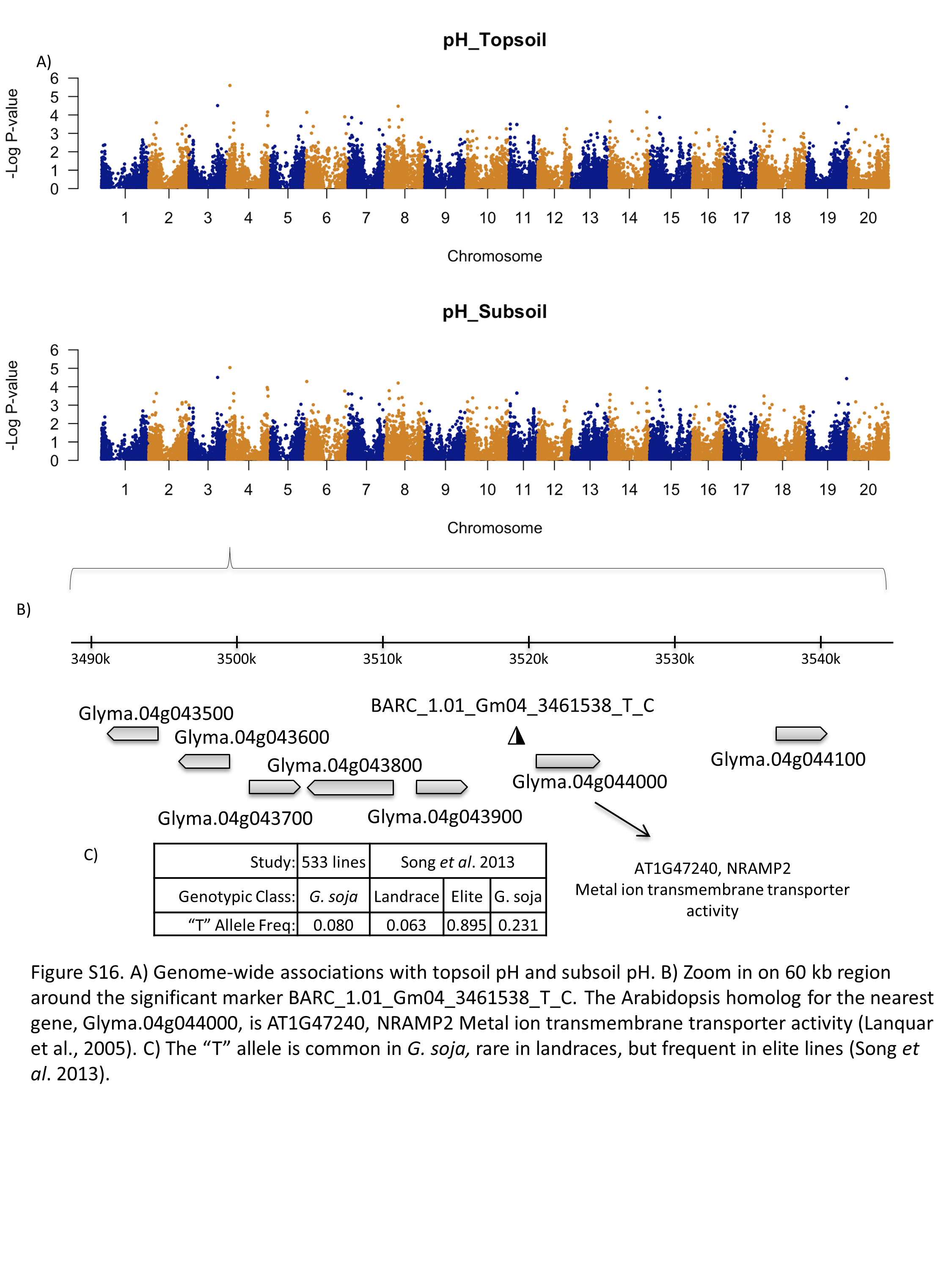

Supplement: Supporting Information [file supp_g3.116.026914_FigureS16.tif]
