# Supplementary material for: Environmental Association Analyses Identify Candidates for Abiotic Stress Tolerance in Glycine soja, the Wild Progenitor of Cultivated Soybeans
Source: G3 (Bethesda). 2016 Jan 27;6(4):835–43. doi: 10.1534/g3.116.026914 (PMC4825654; doi:10.1534/g3.116.026914)

Figure S12. SoySNP50K Bioclimatic and Biophysical association results displayed in Manhattan plots.


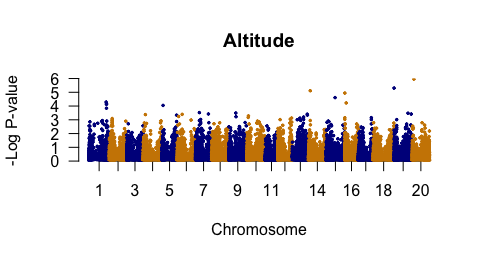

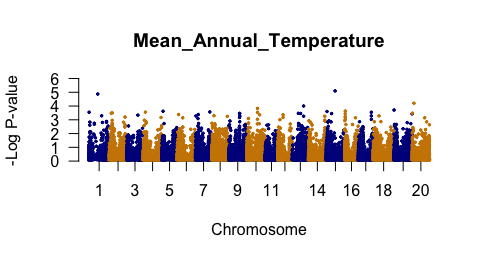

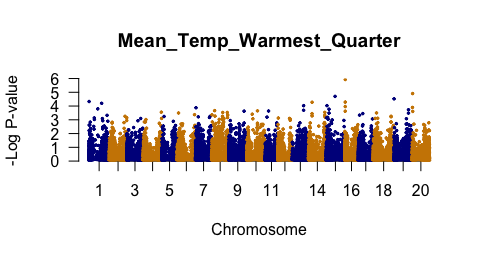

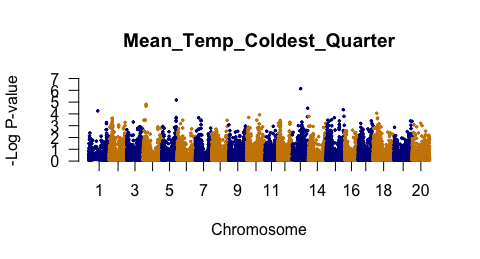

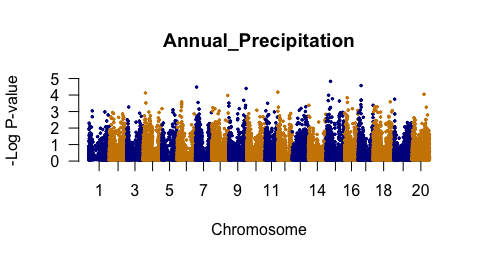

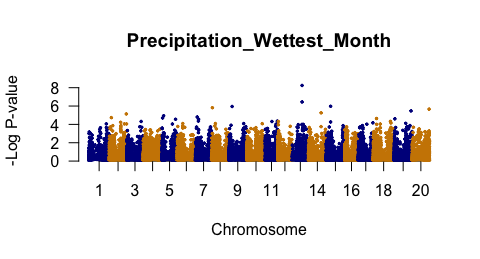

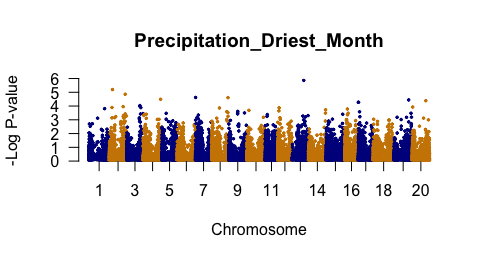

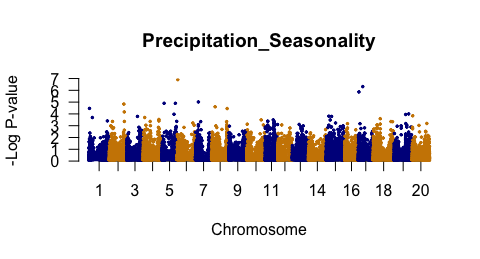

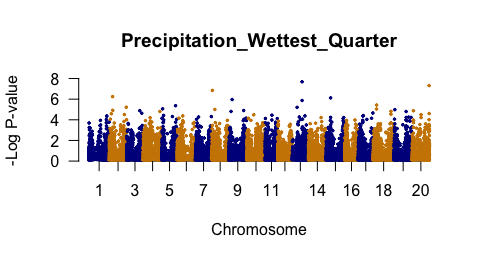

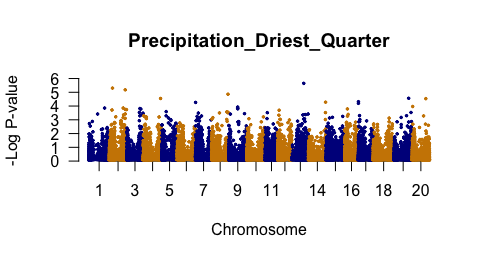

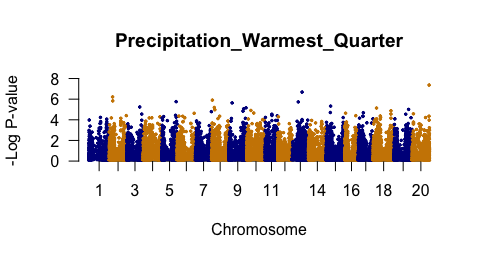

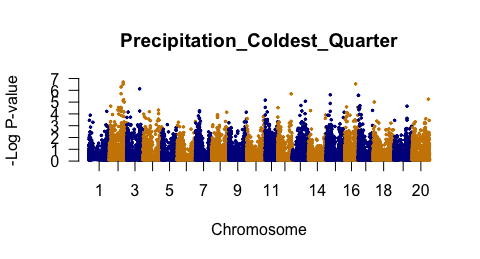

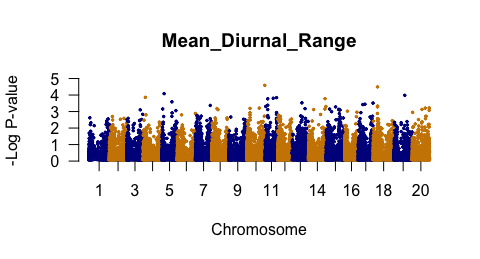

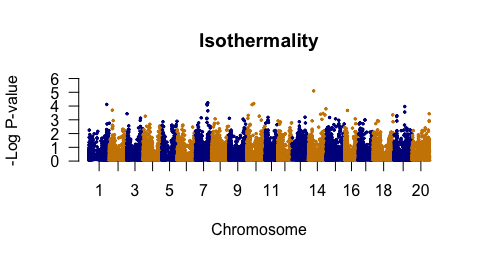

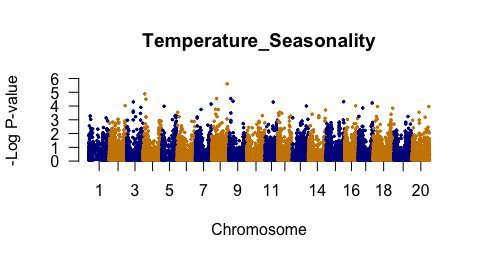

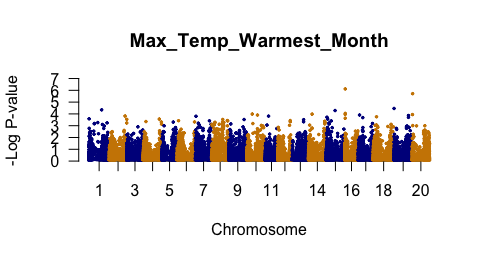

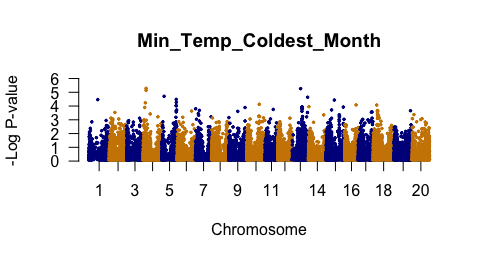

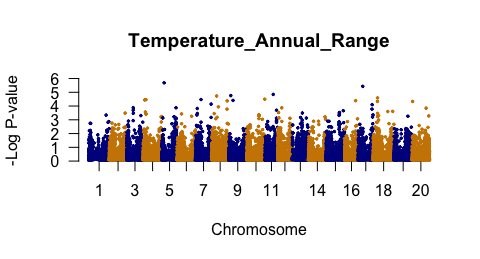

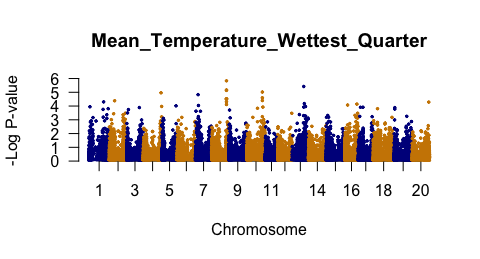

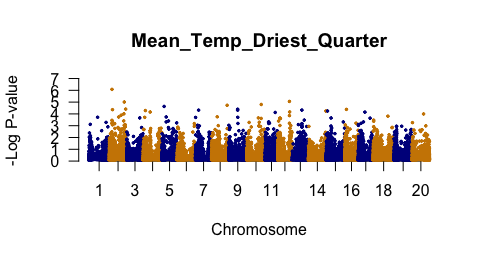

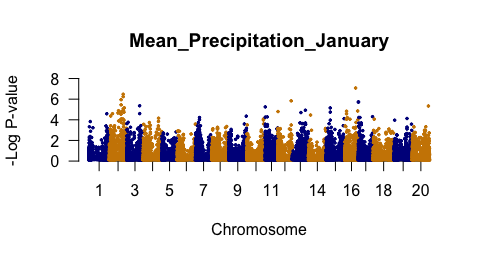

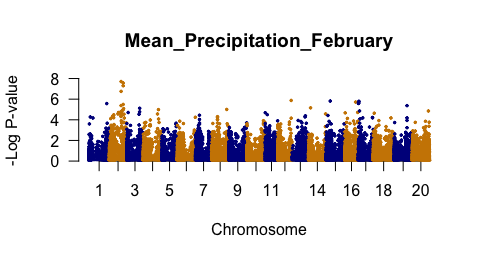

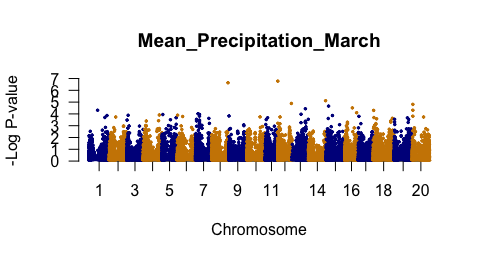

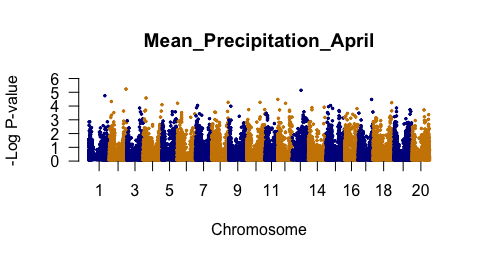

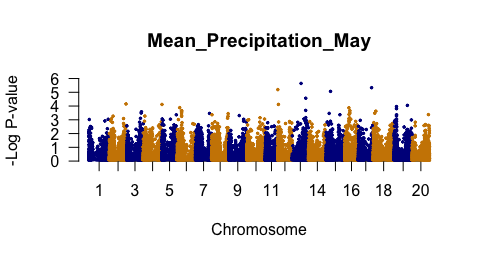

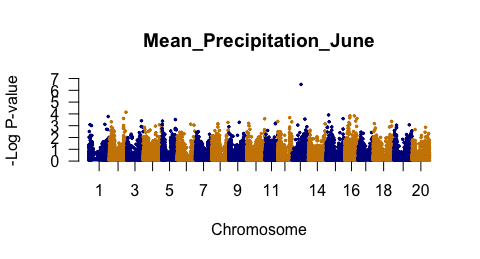

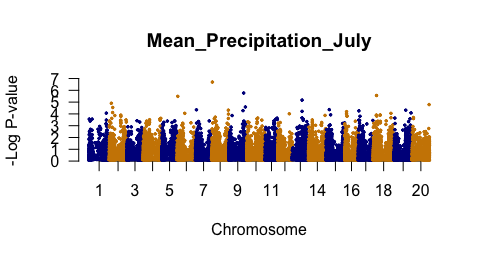

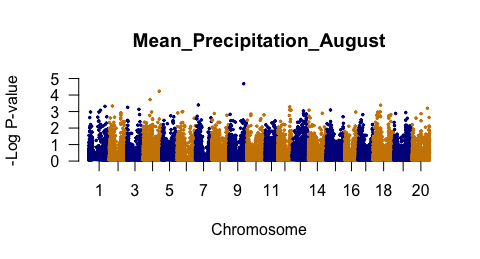

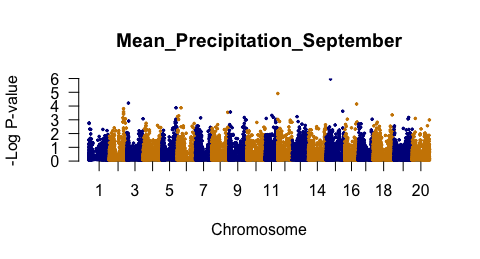

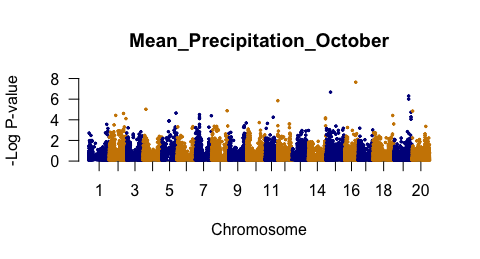

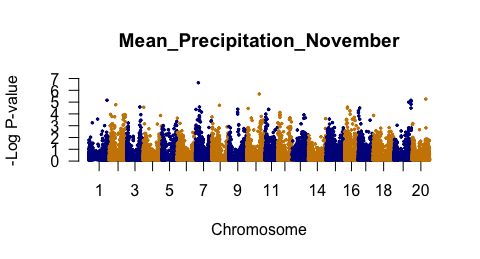

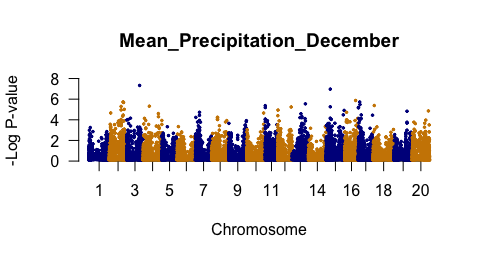

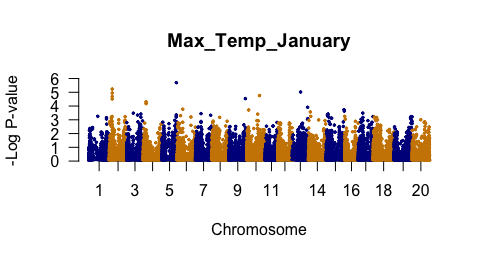

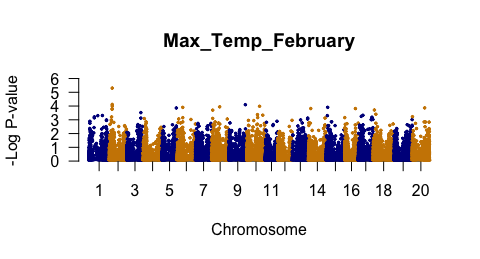

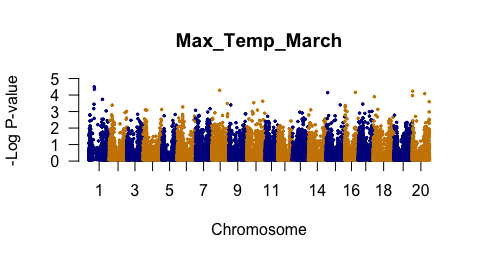

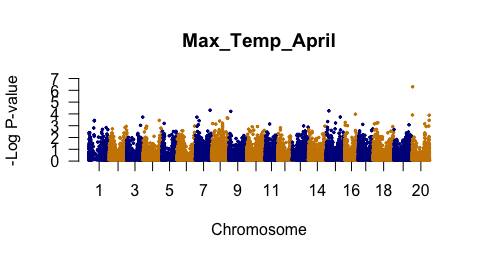

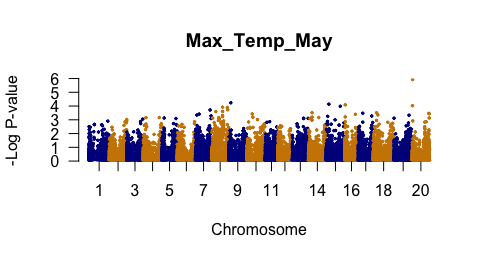

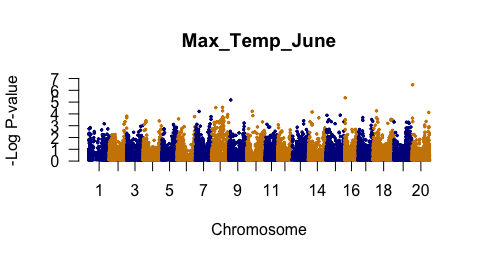

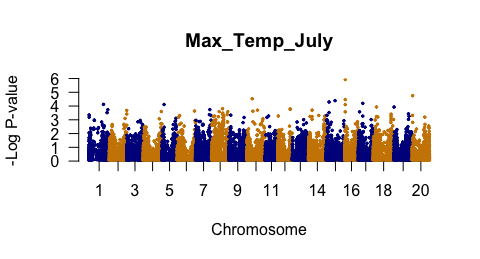

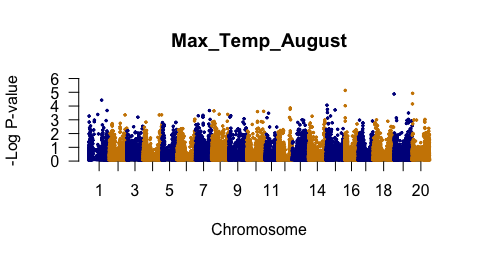

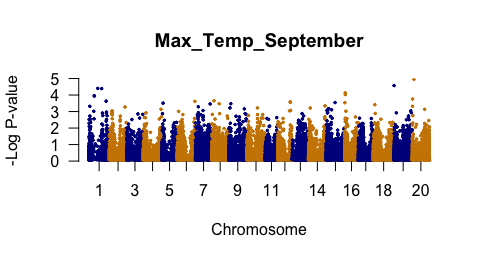

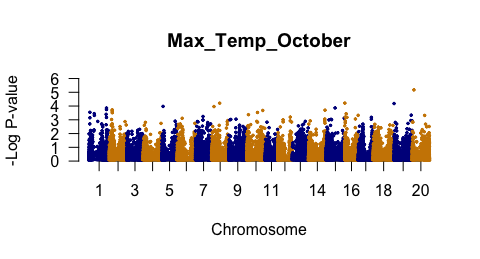

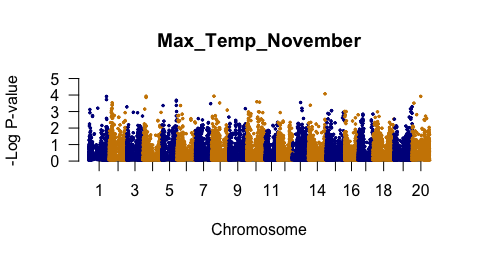

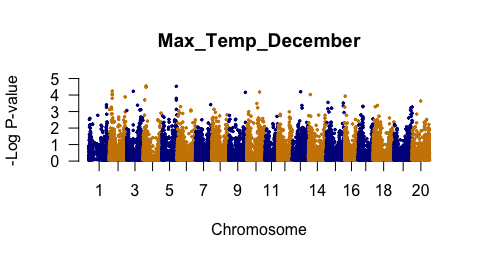

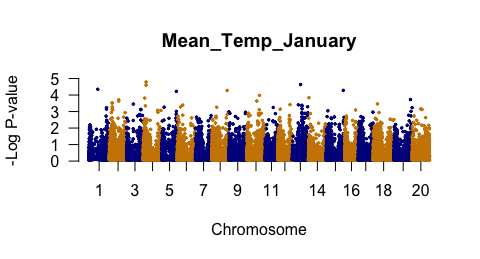

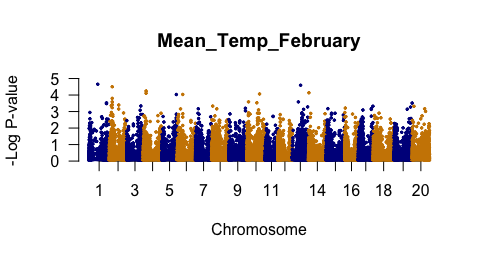

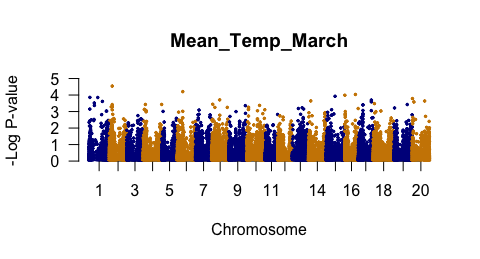

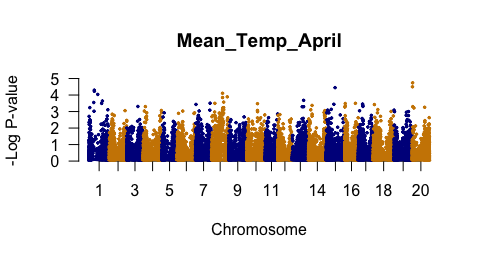

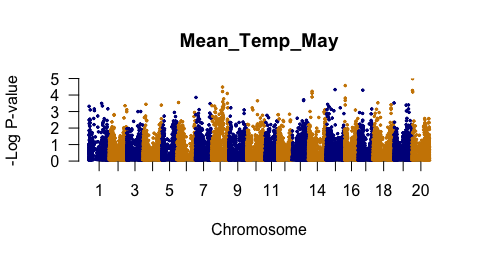

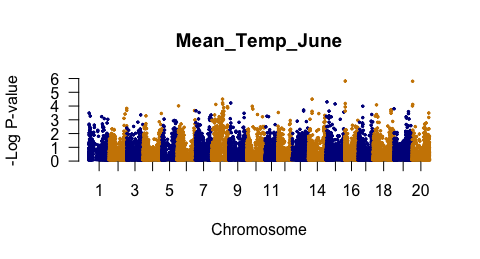

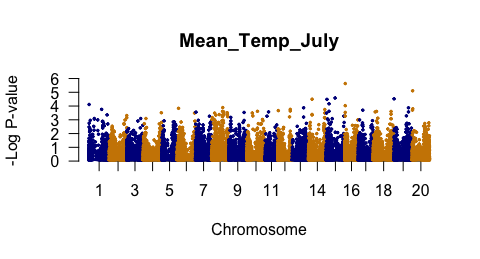

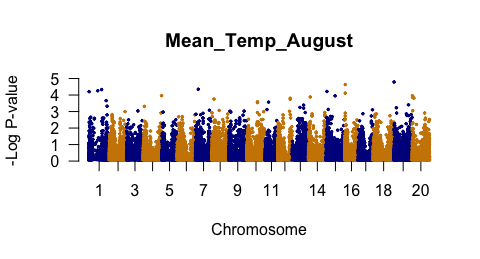

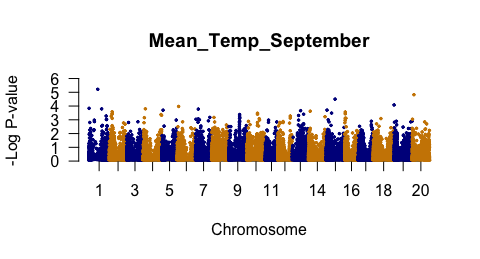

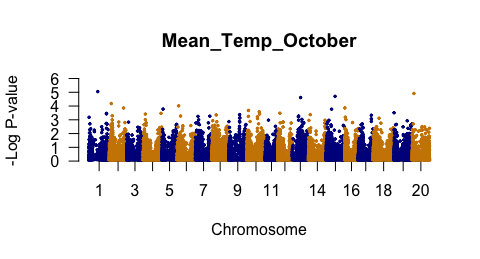

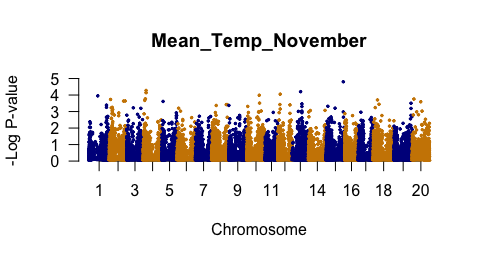

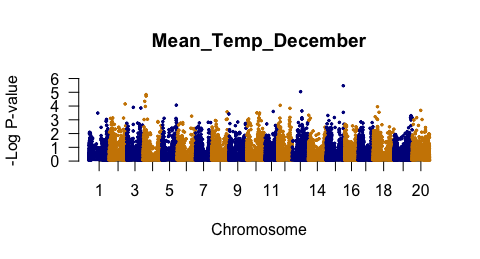

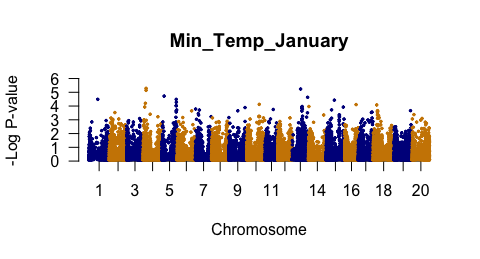

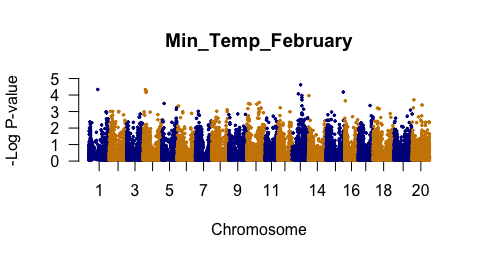

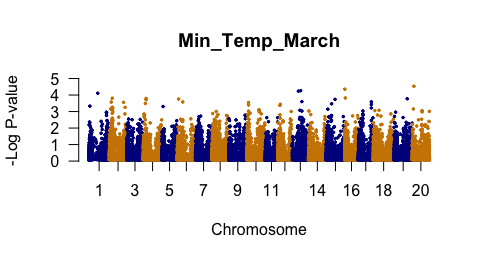

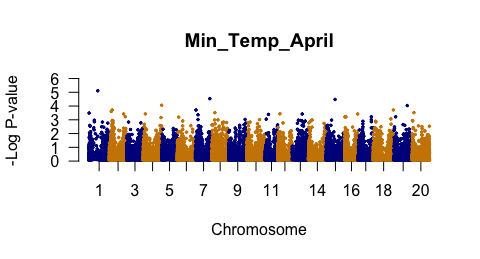

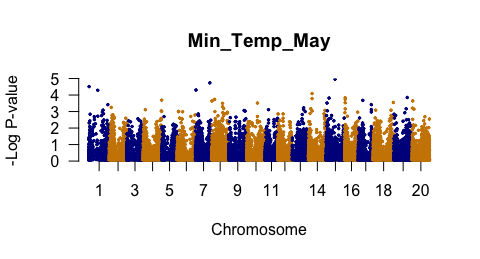

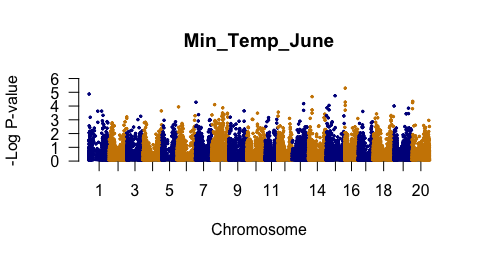

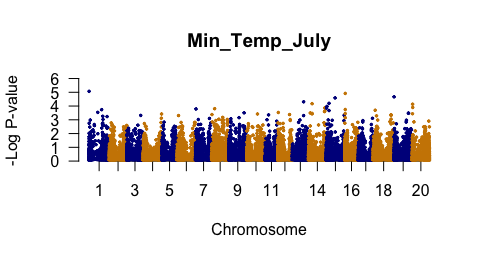

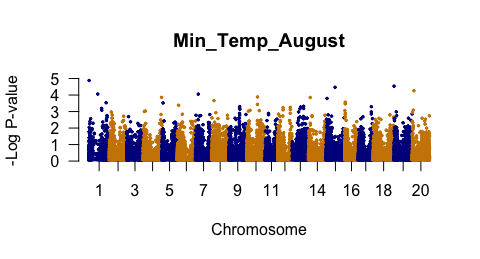

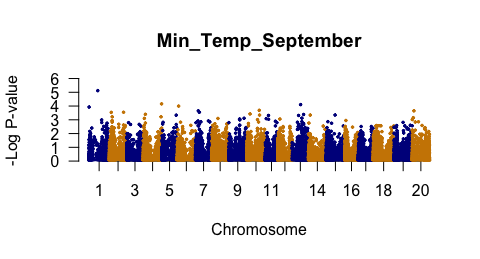

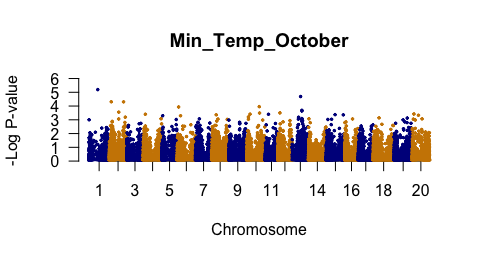

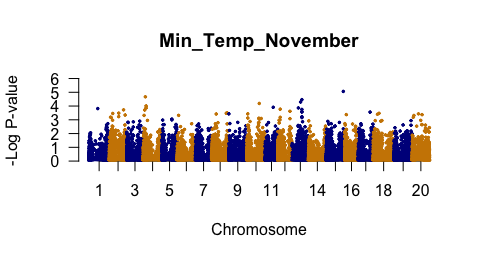

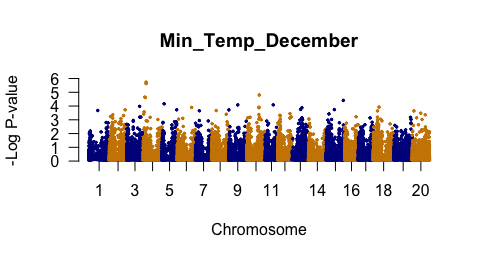

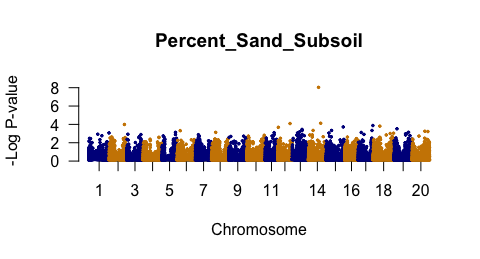

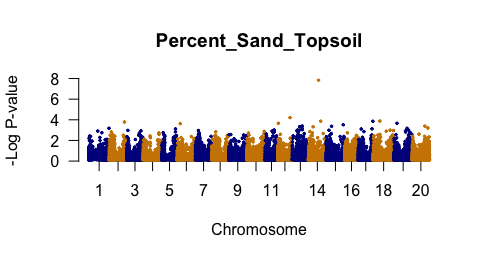

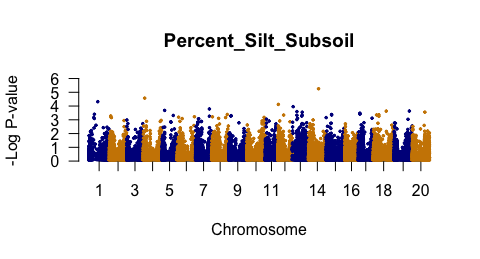

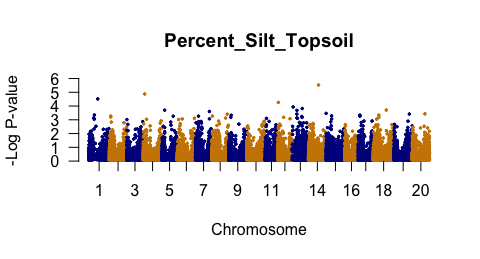

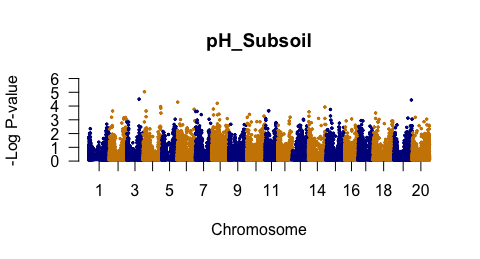

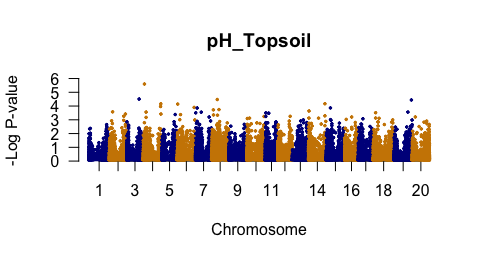

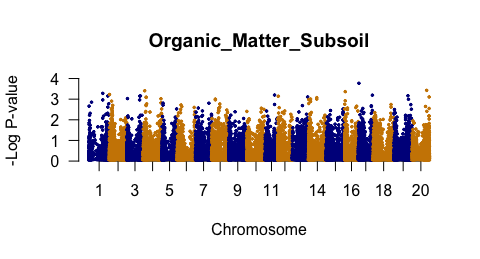

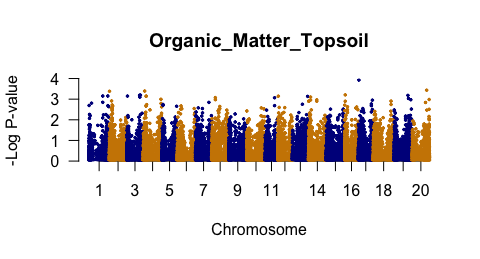

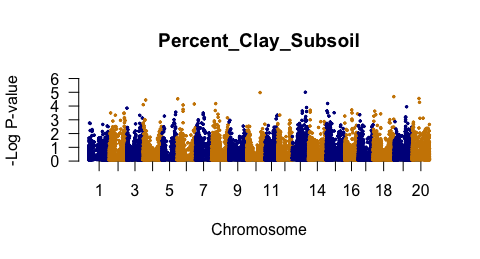

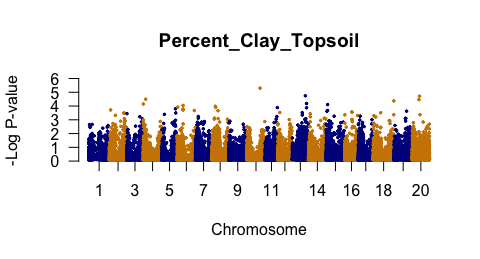

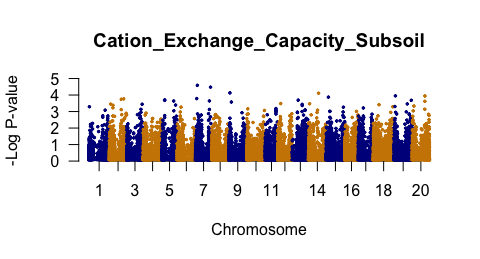

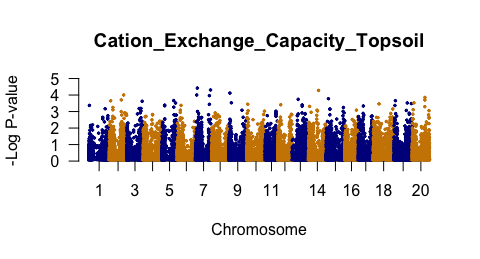

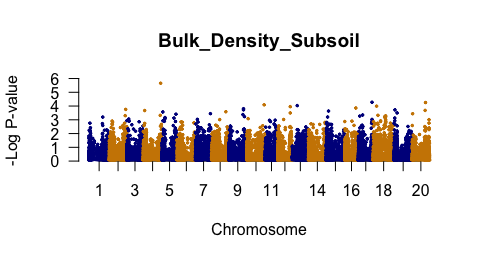

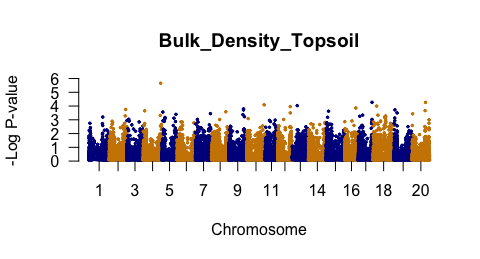

Supplement: Supporting Information [file supp_g3.116.026914_FigureS12.docx]
